# Burden of *Shigella* infection in children under five years living in a peri-urban area of Ouagadougou, Burkina Faso: A community-based cohort study

**DOI:** 10.1371/journal.pntd.0014624

**Published:** 2026-08-14

**Authors:** Alimatou Héma, Jean Sawadogo, Ben Idriss Soulama, Denise Hien, Amidou Diarra, Samuel S. Sermé, Amidou Z. Ouédraogo, Issa Nébié, Sophie Houard, Alphonse Ouédraogo, Alfred B. Tiono, Mahamoudou Sanou, Sodiomon B. Sirima

**Affiliations:** 1 Groupe de Recherche Action en Santé (GRAS), Ouagadougou, Burkina Faso; 2 Université Joseph KI-ZERBO (UJKZ), Ouagadougou, Burkina Faso; 3 European Vaccine Initiative (EVI), Heidelberg, Germany; University of Maryland School of Medicine, UNITED STATES OF AMERICA

## Abstract

**Background:**

*Shigella* is a leading cause of childhood diarrheal disease and a priority target for vaccine development, particularly in low-resource settings where its true burden is often underestimated. A clear understanding of local *Shigella* epidemiology and risk factors is essential for guiding control strategies and vaccine introduction.

**Methodology/Principal Findings:**

We conducted a 12-month longitudinal cohort study enrolling 750 children under five years of age in a peri-urban area of Ouagadougou, Burkina Faso, from December 2020 to March 2021, with follow-up through March 2022. Active and passive surveillance captured both symptomatic and asymptomatic infections. Stool samples (n = 2,401), including 236 from diarrheal episodes, were analyzed by conventional microbiological methods with species confirmation using the BD Phoenix M50 system. *Shigella* was isolated from 56 incident cases, yielding an incidence rate of 6.8 cases per 1,000 child-months (95% CI: 5.3-8.9). *Shigella flexneri* predominated (58.9%), followed by *S. boydii* (23.2%), *S. sonnei* (14.3%), and *S. dysenteriae* (3.6%). Incidence among diarrheal cases was 39.2 per 1,000 child-months (95% CI: 24.7-62.2), alongside substantial asymptomatic carriage (4.9 per 1,000 child-months). Shigellosis was strongly associated with diarrhea, predominantly mucoid rather than bloody stools. Young age was a significant predictor, particularly at 12–23 months (aHR = 2.68; 95% CI: 1.11-6.47) and 36–47 months (aHR = 2.81; 95% CI: 1.08-7.33), while use of a public water source was independently protective (aHR = 0.52; 95% CI: 0.29-0.94).

**Conclusions/Significance:**

This study provides foundational incidence and serotype data for *Shigella* among young children in peri-urban Burkina Faso, informing future vaccine development and targeted public health interventions. The findings highlight the need to strengthen water, sanitation, and hygiene programs, improve diagnostic capacity, and enhance antimicrobial resistance surveillance to reduce *Shigella* transmission and impact in this population.

## Author summary

*Shigella* bacteria are a major cause of diarrheal illness in young children living in resource-limited settings, and they remain an important target for future vaccines. Over one year, we followed 750 children under five years old in a peri-urban community near Ouagadougou, Burkina Faso, through both routine home visits and clinic-based monitoring, testing more than 2,400 stool samples. We found that *Shigella* caused about 7 new infections for every 1,000 months of observation, and nearly 8% of episodes of diarrhea were linked to this bacterium. Most infected children had loose, mucus-containing stools rather than the bloody diarrhea often associated with *Shigella*, meaning many cases could be missed by clinicians looking only for classic symptoms. One species, *Shigella flexneri*, accounted for almost 60% of infections. Younger children and those without access to a public water source were at greater risk. Because standard laboratory culture likely misses many true infections, our findings point to the need for better diagnostic tools, vaccines matched to locally circulating strains, and continued water and sanitation improvements to protect children in similar settings.

## Introduction

Acute diarrheal diseases represent a significant global public health challenge, causing an estimated 1.17 million deaths annually worldwide, with the highest mortality burden observed among children under five years of age, especially in low-income countries. The World Health Organization (WHO) identifies diarrheal disease as a significant health threat, responsible for a substantial proportion of deaths among children under five years of age, particularly in low- and lower-middle-income countries. Sub-Saharan Africa and South Asia bear the greatest burden of these deaths, reflecting ongoing disparities in sanitation, nutrition, and healthcare access worldwide [1].

*Shigella* is the second most common pathogen responsible for diarrhea infectious. Diarrhea caused by *Shigella*, known as shigellosis, is an acute invasive enteric infection manifested mainly by bloody diarrhea. *Shigella* belongs to the *Enterobacterales* family and comprises four distinct serogroups: *Shigella dysenteriae, Shigella flexneri*, *Shigella boydii* and *Shigella sonnei*. The geographical distribution of these species varies according to socio-economic level [2]. *Shigella flexneri* predominates in developing countries, while *Shigella sonnei* is more common in developed countries [2,3]. On the other hand, although *Shigella dysenteriae* and *Shigella boydii* are more often found in developing countries, globally they are more rarely isolated compared to *Shigella flexneri* [4].

Shigellosis is one of the most prevalent diarrheal diseases in developing countries [3]. It contributes to substantial morbidity, with an estimated over 267 million cases annually, nearly 69% of which occur in children under five, particularly in developing countries, with approximately 64,000 deaths in children under five years each year [5]. In these children, shigellosis can also impair psychomotor development [6], and contribute to linear growth faltering [7–9], even in asymptomatic carriers. The persistence of *Shigella* after diarrheal episodes often leads to asymptomatic carrier states, complicating efforts to reduce shigellosis [9]. Numerous studies have documented a high number of asymptomatic *Shigella* carriers in developing countries, perpetuating the cycle of the infection [8,9].

In endemic areas, the lack of adequate latrines, poor hygiene, and limited access to clean drinking water contribute to the spread of *Shigella* [10]. Climatic factors and unfavorable socio-economic conditions, particularly in sub-Saharan Africa’s tropical climate, further facilitate its transmission [11]. Given these challenges, vaccination remains one of the most effective tools of prevention [12–14]. However, comprehensive data on *Shigella* epidemiology are essential for designing effective vaccines and guiding vaccination policies [15].

The deficiency in technical capacity for surveillance in many developing countries, including Burkina Faso, contributes to a substantial underestimation of the burden of shigellosis [5]. Moreover, limited data on shigellosis in community settings, with a predominant focus on hospital studies [10,16,17], further hinder an accurate representation of the true burden and diversity of *Shigella*.

The management of shigellosis is further complicated by the increasing threat of antimicrobial resistance (AMR) due mainly to misuse, overuse or abuse of antibiotics [18]. This misuse of antibiotics accelerates the development of drug-resistant pathogens, making infections harder to treat and control. AMR in *Shigella* has become a significant concern, as resistant strains are associated with higher rates of treatment failure, prolonged illness, and increased mortality. The WHO has prioritized *Shigella* as a critical target for new antimicrobial therapies and interventions due to this growing resistance [19,20].

In developing countries, the misuse of antibiotics is often driven by factors such as inadequate healthcare infrastructure, lack of regulation, and easy over-the-counter access to antibiotics without prescriptions [21]. Additionally, the overuse of antibiotics in agriculture and animal husbandry contributes to the development and spread of resistant strains. As a result, the treatment options for shigellosis are becoming increasingly limited, posing a severe public health threat [22].

Enhanced surveillance capacity improves the detection and estimation of shigellosis, leading to more appropriate vaccine policies and antimicrobial stewardship programs.

This study is one of the objectives of the ShigaPlexIM consortium, which aims to characterize the epidemiology of *Shigella* infections across multiple African sites. As a component of this effort, we conducted a longitudinal baseline epidemiological study in peri-urban areas of Ouagadougou, Burkina Faso, to estimate the incidence of *Shigella* infections (symptomatic and asymptomatic) among children under five years of age. Secondary objectives included characterizing the circulating *Shigella* serogroups and identifying key risk factors associated with the burden of infection within this population.

## Methods

### Ethics statement

The study was approved by the Health Research Ethics Committee (HREC) of Burkina Faso, with the project reference number: 2020-3-055 dated 25 March 2020, Protocol Version: 1.0, February 2020 ShigOraVax (NCT04312906). Written informed consent was obtained from the children’s parents or guardians at the time of enrolment for participation in the study and the storage of samples for future use. The anonymity and confidentiality of the information collected were preserved by assigning unique identification numbers, so as not to infringe on the dignity of the participants. Information provided by participants was kept confidential and password protected.

### Study site

The study was conducted in a peri-urban community on the outskirts of Ouagadougou, Burkina Faso’s capital city. Located in the Sahel region, Burkina Faso covers a total area of 274,200 km^2^. Ouagadougou, the capital city, is located in the central region of the country, which is characterized by the highest population growth rate 4.42% [23]. The specific study area included the neighborhoods of Polesgo and Nioko 2, both unplanned growth zones situated near the Kossodo industrial zone [24]. These neighborhoods fall within the Kossodo health district.

**Fig 1:**
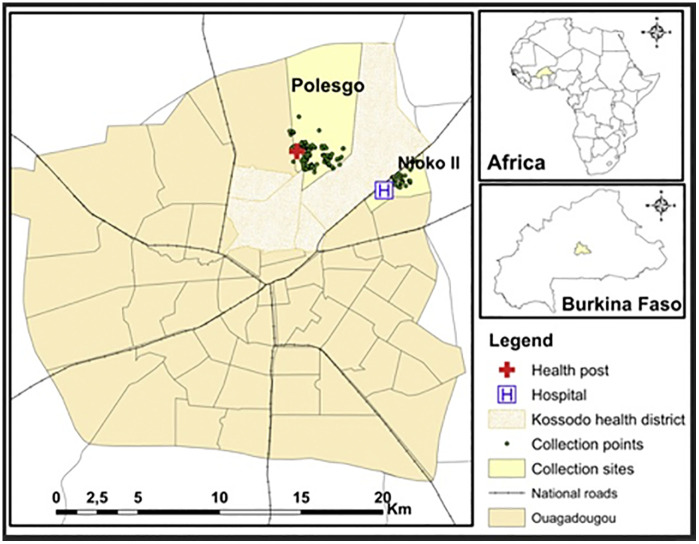
Map of Ouagadougou city showing the study sites with neighborhood boundaries (left), the map of Africa and the map of Burkina Faso (right). Note on Map Attribution and Data Sources: Base layer link: https://www.arcgis.com/apps/mapviewer/index.html?panel=gallery&layers=f9138401ef6f4f0c878489880ac27529&catalog=e3efa3e9a3484eed8b2292adeb736015 Underlying boundary/shapefile source: https://data.humdata.org/dataset/cod-ab-bfa (Burkina Faso - Subnational Administrative Boundaries, OCHA Humanitarian Data Exchange). License/ terms of use: Except where otherwise noted, content on the Humanitarian Data Exchange (HDX) platform, including this dataset, is licensed under a Creative Commons Attribution 4.0 International license (CC BY 4.0), as stated at https://data.humdata.org/dataset/cod-ab-bfa. Visualization Author: MOUNGOU Abakoudjiga Arsene Geographic Data Source: Base Nationale de Données Topographiques (BNDT 2016). Software: ArcGIS version 10.8.

The surroundings of Ouagadougou, akin to many outskirts of African capital cities, consist of informal settlements where the most vulnerable populations reside [25]. The urban planning and environmental services in these peri-urban areas are inadequate, marked by high housing density, poor road networks, a lack of access to clean drinking water, and insufficient sanitation measures. As of June 2010, these two areas comprised approximately 37,878 households [24]. The challenging living conditions in these peri-urban areas underscore the importance of studying the incidence of *Shigella*-associated diarrheal diseases to inform public health interventions and improve the well-being of these communities. Fig 1 provides a detailed map of the peri-urban study area, showing the geographical locations of the Polesgo and Nioko 2 neighborhoods, key landmarks, and the healthcare facilities involved in participant recruitment.

### Study design and participants

This research formed part of the ShigaPlexIM consortium. Due to logistical constraints during the COVID-19 pandemic affecting diagnostic standardization across sites, this manuscript reports the site-specific baseline epidemiology for Burkina Faso. Molecular analysis (RLDT) on a subset of these samples has been reported elsewhere [26].

The present manuscript presents data from the Burkina Faso site, where 750 children under five years were enrolled between December 2020 and March 2021 and followed for 12 months. The overall study period spanned up to 16 months (December 2020 to March 2022), reflecting the enrollment phase followed by individual 12-month follow-up for each child post-enrollment.

The study was conducted in the Nongr-Massom health district of Ouagadougou, encompassing the peri-urban neighborhoods of Polesgo (six quartiers) and Nioko 2 (eight zones). Recruitment was performed through a standardized community-based procedure aligned with the study’s Standard Operating Procedure (SOP). Community Health Workers collaborated closely with local health authorities, community leaders, and residents to facilitate engagement and mobilization.

Households with children under five years were identified by Community Health Workers, who informed families about the study objectives, procedures, and participation benefits. Interested parents or guardians registered their children on a volunteer list and received unique identification code. To ensure equitable representation, quota targets were set at approximately 300 children from Polesgo and 450 from Nioko 2. When volunteer numbers exceeded quotas, a simple random drawing without replacement was conducted publicly to select participants fairly.

Selected families were then invited to attend screening and enrollment visits at designated health facilities: Polesgo Health care and Social Promotion Center (CSPS) for Polesgo residents and Kossodo Medical Center (CMA) for Nioko 2 residents. Screening involved confirmation of eligibility, informed consent procedures, and baseline data collection. Eligible participants were permanent residents without plans to relocate during the subsequent 12-month follow-up. Non-inclusion criteria included children born after recruitment, known or suspected immunodeficiency, major congenital anomalies, recent immunosuppressive therapy, or concurrent participation in other clinical studies.

After enrollment (designated as Month 0, M0), participants attended scheduled visits at the health facilities for epidemiological and clinical data collection. Additionally, unscheduled visits occurred whenever caregivers sought care for a child with diarrheal symptoms at the study health centers (passive surveillance).

Active surveillance included community sensitization to diarrheal symptoms and encouragement to seek prompt care at sentinel health centers, where stool samples were collected, cases were managed, and clinical data recorded.

The last enrolled participant completed follow-up in March 2022, concluding the longitudinal surveillance period.

### Definitions

Diarrhea was defined as three or more loose stools per 24-hour period according to the official WHO guidelines on diarrhea management [27].

A new diarrheal episode was defined as any occurrence of diarrhea beginning after at least seven diarrhea-free days, following the epidemiological case definition established by the Global Enteric Multicenter Study (GEMS) [28]. Any positive stool sample for *Shigella* was defined as an episode of *Shigella* infection. A case of asymptomatic *Shigella* infection was defined as any non-diarrheal stool sample that tests positive for *Shigella* by stool culture. *Shigella* cases were confirmed by phenotyping, including BD Phoenix M50.

Moderate to Severe Diarrhea (MSD) was defined according to the GEMS criteria [28] as a diarrheal episode (≥3 loose stools within 24 hours) at least one of the following: signs of dehydration (sunken eyes or slow/very slow skin pinch recoil); clinician’s assessment requiring intravenous rehydration or hospitalization; or visible blood in the stool (dysentery).

Asymptomatic cases were defined as children seen at the health care center without diarrhea during a planned visit (at screening, month 6 and month 12 visits).

### Sample size calculation

Sample size estimations accounted for the multisite design of the ShigaPlexIM baseline study, which was conducted in Burkina Faso and Zambia. To estimate the incidence of *Shigella* moderate-to-severe diarrhea (MSD) with sufficient power and precision, detecting a minimum of 22 events required a cohort size of 734 children. This sample size yielded a two-sided 95% confidence interval with a precision width of 0.025, assuming a hazard rate (λ) of 0.03 and 97% censoring. Considering an anticipated 2% loss to follow-up, the minimum cohort size was adjusted to 750 children. Accordingly, to ensure adequate statistical power at each site, we planned to recruit 750 children per site in Burkina Faso and Zambia.

## Laboratory methods

### Stool samples collection

Stool samples were collected at the “Centre Medical avec Antenne chirurgicale (CMA)” of Kossodo and the “Centre de Santé et de Promotion Sociale (CSPS)” of Polesgo. For asymptomatic children attending scheduled visits, parents or guardians were provided with sterile containers and instructed by trained study staff to collect stool samples either at home or at the health facility before the visit. For children presenting with diarrhea, trained healthcare personnel collected fresh stool samples directly at the health facility to ensure prompt and quality specimen collection. All stool samples were placed in Cary-Blair transport medium and transported to the Groupe de Recherche Action en Santé (GRAS) bacteriology-virology laboratory at a controlled temperature between 2 and 8°C for microbiological analysis.

### Stool culture and identification of *Shigella* isolates

Stool samples were inoculated on MacConkey (MA) and Xylose Lysine Deoxycholate (XLD) agar and selenite F broth (Liofilchem, Roseto degli Abruzzi, Italy). Inoculated plates were incubated at 35–37°C and examined after 18–24 hours according to conventional stool culture techniques [29]. Subcultures from selenite F broth were performed on Salmonella-*Shigella* agar. Non-lactose fermenting colonies were identified through standard biochemical tests. *Shigella* suspected colonies (small pale colonies on MacConkey and red colonies on XLD) were stabbed in Triple Sugar Iron Agar (TSI), Sulphur Indole Motility Agar (SIM) and Lysine Decarboxylase Citrate Agar (LDC) (Beckton & Dickinson, NJ, USA), and subjected to the urease production test. After these tests, API 20E system biochemical tests (BioMérieux, UK). *Shigella* serogroups were identified using polyvalent antisera (Bio-Rad, France). *Shigella flexneri* sv 2b Gp B ATCC 12022 and *Shigella sonnei* ATCC 25931 served as the controls.

Double confirmation of the *Shigella* diagnosis was performed using the BD Phoenix M50 identification Automated Microbiology system (BD Diagnostic Systems version 2.20.0.0/V6,81A(x-US). The Phoenix identification method uses modified conventional, fluorogenic, and chromogenic substrates. Combination panels NMIC/ID-435 (catalogue no. 449044) were used for both identification and susceptibility testing. The ID side contains 45 wells with dried biochemical substrates and 2 fluorescent control wells. The ID broth was inoculated with bacterial colonies adjusted to 0.5 McFarland standard by using a CrystalSpec nephelometer (BD Diagnostics), according to the manufacturer’s recommendations. The suspension was then poured into the ID side of the Phoenix panel after an aliquot (25 μl) was removed for antimicrobial susceptibility testing (AST). The specimen was logged and loaded into the instrument within the specified timeline of 30 minutes. Quality control and maintenance were performed according to the manufacturer’s recommendations. The results were analyzed using Epicenter data management software version 3.01A (BD Diagnostic Systems) after 16 hours of incubation.

### Statistical analysis

Data were analyzed using Stata software version 17 MP (StataCorp LLC). The burden of *Shigella*-attributable moderate-to-severe diarrhea (MSD) and associated risk factors were estimated using survival analysis techniques.

Categorical variables (e.g., maternal education, water source) were recoded into binary or grouped categories to facilitate analysis. String variables were encoded appropriately for regression modeling. Children’s nutritional status at enrollment was assessed using weight and length measurements with weight-for-age (WAZ), height-for-age (HAZ), and weight-for-height (WHZ) Z-scores were computed according to WHO child growth standards. Undernutrition was defined as WAZ < –2 (underweight); HAZ < –2 (stunted); WHZ < -2 (wasted).

A descriptive analysis summarized baseline characteristics using counts and proportions for categorical variables and means ± standard deviations (SD) for continuous variables. High numbers of missing values for some maternal and household variables were noted; these are addressed with explanations in the Methods and footnotes to Table 1.

**Table 1:** Baseline characteristics of the study participants.

Characteristics	Frequency n (%)
Age categories (months) (n = 750)	
0-11	124 (16.5)
12-23	207 (27.6)
24-35	181 (24.1)
36-47	135 (18.0)
48-59	103 (13.7)
Gender (n = 750)	
Male	387 (51.6)
Female	363 (48.4)
Mean weight at enrolment (Kg)	11.1 ± 2.8
Missing	
Nutritional status at enrolment (n = 745)	
Underweight (WAZ < -2.0)	92 (12.3)
Stunted (HAZ < -2.0)	197(26.3)
Wasted (WHZ < -2.0)	42 (5.6)
Normal (WAZ, HAZ/ WHZ ≥ -2.0)	415 (55.6)
Mothers’ characteristics	
Mother’s Age (n = 750)	
25 years and younger (≤25 years)	214 (28.5)
Over 25 years (>25 years)	536 (71.5)
Mother formal education (n = 671)*	
No formal education	336 (50.1)
Some formal education	335 (49.9)
Mother’s occupation (n = 690)*	
Housewife	340 (49.3)
Income generating activity (informal/formal sector)	350 (50.7)
Mother’s marital status (n = 690)*	
Single	39 (5.7)
Married	651 (94.3)
Mother’s number of children (n = 690)*	
Less than 3 living children	409 (59.3)
3 or more living children	281 (40.7)
Household level factors	
Socioeconomic status of the household (n = 690)	
Poor	250 (36.2)
Middle	211 (30.6)
Rich	229 (33.2)
Water, sanitation and hygiene (WASH) of the household	
Main source of drinking water (n = 690)*	
Piped into house	182 (26.4)
Public tap/Borehole	508 (73.6)
Handwashing habit (n = 689)*	
Regularly wash hand	381 (55.3)
Occasionally	308 (44.7)
Type of improved toilet into the house (n = 690)*	
Improved sanitation	559 (81.0)
Unimproved sanitation	131 (19.0)

*Note: The total number of participants is n = 750. Denominators less than 750 for maternal and household variables (e.g., Mother’s formal education, n = 671; Mother’s occupation, n = 690) reflect missing data due to non-response during the baseline socio-demographic interview. All percentages for these characteristics are calculated based on the number of valid responses for each specific item.

The primary outcome was the time to the first episode of *Shigella*, defined by stool culture positivity. Time-at-risk was calculated in child-months for all participants, including those asymptomatic or not attending sentinel health care centers, by summing individual exposure periods until infection or censoring. Incidence rates were computed as the number of first-episode cases divided by the total child-months at risk, expressed per 1,000 child-months.

Predictors of time-to-first *Shigella* infection were assessed using Cox proportional hazards regression. Univariable (bivariate) models were first applied for initial screening to identify potential risk factors associated with *Shigella* infection. Subsequently, all relevant explanatory variables were entered into multivariable models to estimate adjusted hazard ratios (HRs), controlling for confounding factors. For time-varying covariates such as diarrhea episodes, extended Cox models were used.

Variables were selected for the multivariate Cox proportional hazards model based on a univariate screening process where factors with a p-value < 0.20 or known biological relevance (age, sex, and nutritional status) were included.

Hazard ratios (HR) were reported with 95% confidence intervals (CIs). The proportional hazards assumption was checked using Schoenfeld residuals and time-interaction terms, confirming model validity.

Covariates in the analysis included sociodemographic factors (maternal education, occupation), household-level factors (toilet type, water source, waste disposal), and child nutritional status indicators based on weight and length measurements. Weight-for-age (WAZ), height-for-age (HAZ), and weight-for-height (WHZ) Z-scores were calculated according to the World Health Organization (WHO) child growth standards (2006). Undernutrition was defined using standard cutoffs: WAZ < - 2 indicating underweight, HAZ < - 2 indicating stunting, and WHZ < - 2 indicating wasting [30].

## Results

### Study population characteristics

We enrolled 750 under-five years children (51.6% male) in this 12-month study. Participants had a median age of 28.1 months (IQR: 15.0; range: 2–59) with right-skewed distribution (skewness = 22.2). Anthropometric measures showed mean weight 11.1 ± 2.8 kg (median: 12.0, IQR: 4.2) and height 89.8 ± 37.3 cm (median: 88.8, IQR: 17). Nutritional deficiencies included stunting (26.3%, HAZ < -2), wasting (5.6%, WHZ < -2), and underweight (12.3%, WAZ < -2). Most mothers were >25 y ears (71.5%), married (94.4%), with 50.1% having no formal education and 49.3% unemployed. The characteristics of the study participants and their households are described in Table 1.

### *Shigella* incidence

**Fig 2:**
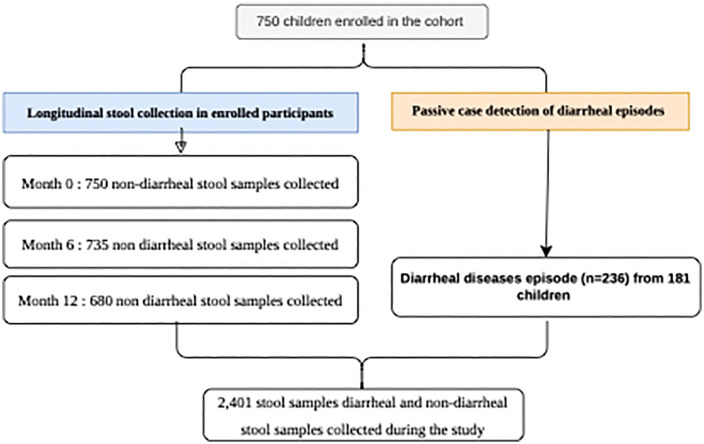
Study flowchart. This flowchart illustrates participants enrollment and stool sample collection during a twelve-month cohort study in peri-urban area of Ouagadougou, Burkina Faso. A total of 750 children were enrolled and provided non-diarrheal stool samples at baseline (Month 0), Month 6, and Month 12. Passive case detection identified 236 diarrheal episodes from 181 children during follow-up. In total, 2,401 stool samples (236 diarrheal and 2,165 non-diarrheal) were collected and tested for Shigella using culture methods. Note: Scheduled visits: planned surveillance visits at the health care facility (including Month 0, Month 6, and Month 12) for routine clinical assessment and collection of non-diarrheal stool samples. Unscheduled visits: visits initiated by caregivers when a child presented to the health care facility with acute diarrhea (passive case detection).

During the twelve-month follow-up period, a total of 2,401 stool samples (236 diarrheal and 2,165 non-diarrheal samples) were tested for *Shigella* by culture as described in the flowchart (Fig 2).

A total of 236 diarrheal episodes were recorded during the follow-up period. Based on the Vesikari Clinical Severity Scoring system, 225 (95.3%) of these episodes were classified as Moderate-to-Severe Diarrhea (MSD), with 113 episodes categorized as severe and 112 as moderate.

From all stool samples, 76 cases of *Shigella* were isolated, of which, sixteen cases of *Shigella* had occurred, after the first incident episode, and therefore were not included in the analysis.

A total of 60 incident cases of confirmed Shigella were identified. Because four participants experienced their episode on the day of enrollment, they were excluded from longitudinal risk calculation, leaving 56 incident cases for the primary analysis, yielding an incidence rate of *Shigella* infections of 6.8 episodes (95% CI: 5.3, 8.9) per 1000 child-months (Table 2). The incidence rates of *Shigella* infections according to sex were 7.1 (95% CI: 4.9, 10.3) and 6.5 (95% CI: 4.5, 9.5) respectively for females and males. According to age groups, the highest incidence of overall *Shigella* infections occurred in children aged between 12–23 months with 10.0 episodes per 1000 child-months. The incidence rate of asymptomatic cases of *Shigella* was 4.9 (95% CI: 3.6, 6.8) per 1000 child-months and the incidence of diarrheal cases was 39.2 (95% CI: 24.7, 62.2)

**Table 2:** Incidence of *Shigella* infection in children under five years of age.

Child characteristics	Incidence of *Shigella* (95% CI) per 1000 child-months
Overall	6.8 (5.3, 8.9)
Type of stools	
Diarrheal	39.2 (24.7, 62.2)
No-diarrheal	4.9 (3.6, 6.8)
Age groups (months)	
0-11	6.4 (3.3, 12.3)
12-23	10.0 (6.6, 15.2)
24-35	4.0 (2.0, 8.0)
36-47	8.7 (5.1, 15.1)
48-59	3.5 (1.3, 9.3)
Sex	
Female	7.1 (4.9, 10.3)
Male	6.5 (4.5, 9.5)
Nutritional status at enrollment (z-score)	
Underweight (WAZ < -2.0)	
Yes	3.0 (1.0, 9.4)
No	7.4 (5.6, 9.6)
Stunting (HAZ < -2.0)	
Yes	4.6 (2.5, 8.6)
No	7.6 (5.7, 10.1)
Wasting (WHZ < -2.0)	
Yes	6.5 (2.1, 20.0)
No	6.9 (5.2, 9.0)
Diarrheal characteristics	
Diarrheal severity (Vesikari score)	
Mild	–
Moderate	28.1(14.0, 56.2)
Severe	47.2 (25.4, 87.7)
Mucus in stools	
Yes	46.3 (27.4, 78.2)
No	23.2 (8.7, 61.7)
Blood in stools	
Yes	105.1 (39.5, 280.2)
No	30.1 (17.8, 50.8)
Fever	
Yes	58.2 (27.8, 122.2)
No	28.7 (15.9, 51.8)
Vomit	
Yes	35.7 (13.4, 95.1)
No	35.7 (21.2, 60.3)
Water, sanitation and hygiene (WASH)	
Household water source	
Piped into house	9.9 (6.4, 15.3)
Public tap/borehole	6.3 (4.6, 8.8)
Regularly wash hand	
Yes	9.0 (6.5, 12.3)
No	5.2 (3.3, 8.2)
Type of toilet into the house	
Yes	7.6 (5.8, 10.1)
No	5.1(2.3, 11.4)

per 1000 child-months (Table 2). Of the cases of diarrhea, the incidence estimate by diarrhea severity was 28.1 (95% CI: 14.0, 56.2) for moderate cases and the incidence of the severe cases was 47.2 (95% CI: 25.4, 87.7).

### Risk factors associated with *Shigella* infection

In the univariate analysis, the type of stool sample (diarrheic or non-diarrheic) was identified as the most crucial factor associated with *Shigella* infection. Indeed, having diarrhea showed the strongest association with *Shigella* infection (Hazard Ratio [HR] = 13.52 95% CI: 7.18, 25.45; p < 0.001) compared with asymptomatic cases. Individuals with diarrheic stools were thirteen times more likely to have *Shigella* infection compared to those with non-diarrheic stools. This underscores the importance of *Shigella* as a causative agent of diarrhea.

In the multivariate analysis, child age categories emerged as significant predictors of infection. Compared to infants (0–11 months), children in the 12–23-month age group (Adjusted Hazard Ratio [aHR] = 2.68; 95% CI: 1.11, 6.47; p = 0.033) and the 36–47-month age group (aHR = 2.81; 95% CI: 1.08, 7.33; p = 0.040) exhibited significantly higher risks of *Shigella* infection. Stunting showed a non-significant protective trend in both the univariate (HR = 0.59; 95% CI: 0.28, 1.24; p = 0.181) and multivariate models (aHR = 0.58; 95% CI: 0.26, 1.31; p = 0.199).

Among household-level WASH variables, using public water sources showed a borderline significant protective effect in the univariate analysis (HR = 0.63; 95% CI: 0.38, 1.06; p = 0.076), which became statistically significant after adjusting for confounders in the multivariate model (aHR = 0.52; 95% CI: 0.29, 0.94; p = 0.031). Other WASH factors displayed non-significant protective trends in the univariate analysis, including having a covered water container (HR = 0.73; 95% CI: 0.32, 1.68; p = 0.404), utilizing improved toilet facilities (HR = 0.64; 95% CI: 0.29, 1.46; p = 0.270), and regular handwashing (HR = 0.68; 95% CI: 0.39, 1.18; p = 0.150). Open defecation showed no notable association with infection (HR = 1.05; 95% CI: 0.51, 2.18; p = 0.800).

Other participant-level demographic and clinical variables showed limited associations. Gender showed no significant link to infection (HR = 1.14; 95% CI: 0.68, 1.91; p = 0.682), and wasting demonstrated no association (HR = 0.92; 95% CI: 0.30, 2.82; p = 0.835). Socioeconomic factors, including maternal formal education (HR = 1.45; 95% CI: 0.84, 2.51; p = 0.183) and household wealth index (p = 0.679 for the overall univariate test), were not significantly associated with the outcome in univariate models. However, in the multivariate model, the overall effect of household wealth became significant (p = 0.025), driven by a higher risk within the “Fair” wealth category (aHR = 2.28; 95% CI: 1.11, 4.68; p = 0.025).

The analysis confirms that diarrheic stool type is by far the strongest predictor of *Shigella* infection across both univariate and multivariate assessments. While several WASH-related factors showed suggestive protective trends, only the use of public water source reached independent statistical significance in the final multivariable model (Table 3).

**Table 3:** Risk factors associated with *Shigella* infection in children under five years in peri-urban area of Ouagadougou, Burkina Faso.

Variable	Univariate analysis	p-value	Multivariate analysis	p-value
Diarrheal stool (Ref: Non-diarrheal)	13.52 (7.18, 25.45)	<0.001	15.11 (7.51, 30.42)	<0.001
Child characteristics				
Age groups (Ref: 0–11 months)		0.081		0.035*
12–23 months	1.61 (0.73, 3.54)	0.244	2.68 (1.11, 6.47)	0.033
24–35 months	0.58 (0.21, 1.61)	0.343	1.25 (0.43, 3.63)	0.660
36–47 months	1.41 (0.60, 3.32)	0.417	2.81 (1.08, 7.33)	0.040
48–59 months	0.59 (0.18, 1.92)	0.390	1.18 (0.32, 4.33)	0.771
Female sex (Ref: Male)	1.14 (0.68, 1.91)	0.682	1.07 (0.60, 1.92)	0.815
Stunted (Ref: Not stunted)	0.59 (0.28, 1.24)	0.181	0.58 (0.26, 1.31)	0.199
Wasted (Ref: Not wasted)	0.92 (0.30, 2.82)	0.835	0.95 (0.30, 3.02)	0.852
Mother’s education (Ref: None)				
Some formal education	1.45 (0.84, 2.51)	0.183	1.46 (0.81, 2.62)	0.208
Household characteristics				
Socioeconomic status (Ref: Poor)		0.679		0.025
Fair	1.32 (0.66, 2.42)	0.446	2.28 (1.11, 4.68)	0.025
Rich	1.01 (0.51, 1.94)	0.977	2.12 (0.96, 4.67)	0.059
Water, sanitation, and hygiene (WASH)				
Main water source (Ref: Other)				
Public water source	0.63 (0.38, 1.06)	0.076	0.52 (0.29, 0.94)	0.031
Regular handwashing (Ref: No)	0.68 (0.39, 1.18)	0.150	0.77 (0.41, 1.46)	0.434
Toilet type (Ref: Improved sanitation)	0.64 (0.29, 1.46)	0.270	0.71 (0.31, 1.65)	0.415
Covered water container (Ref: No)	0.73 (0.32, 1.68)	0.404	0.66 (0.21, 2.07)	0.492
Open defecation (Ref: No)	1.05 (0.51, 2.18)	0.800	1.06 (0.49, 2.29)	0.913

*Age category overall significance in univariate (p = 0.081) but significant subgroups in multivariate. For the variable “Toilet type”, the reference category is “Improved sanitation.” Hazard ratios (HR) represent the relative risk of Shigella infection among children living in households with unimproved toilets compared to those with improved sanitation facilities.

### Proportional hazards assessment

**Fig 3:**
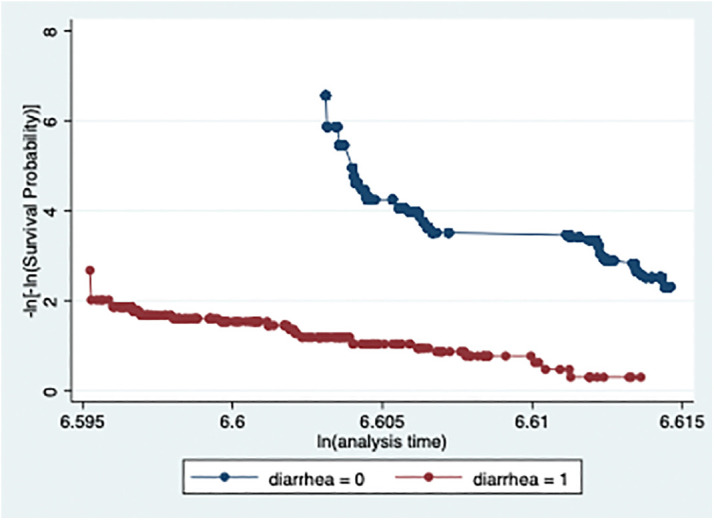
Log-log survival curves by diarrhea status. Parallel curves suggest proportional hazards are met for diarrhea (0 = absent, 1 = present). The vertical distance reflects the constant hazard ratio (HR = 13.5) over time.

Visual inspection of log-log survival curves demonstrated parallel trajectories between groups (Fig 3), supporting the proportional hazards assumption for diarrhea status (log-rank test p = 0.765). The constant vertical separation of curves indicates the hazard ratio remained stable throughout follow-up, with no evidence of time-dependent effects (Schoenfeld residuals test p = 0.812).

### Distribution of *Shigella* species

**Fig 4:**
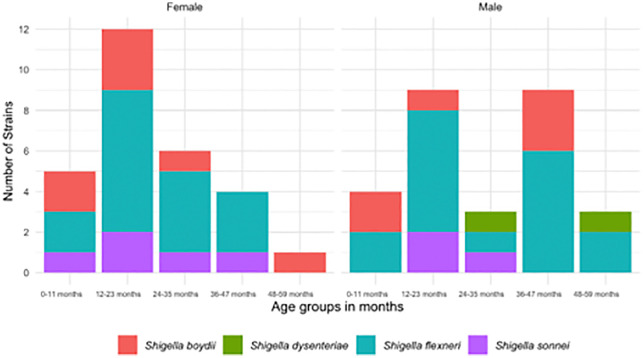
*Shigella* serogroups distribution according to age groups and gender.

All four *Shigella* serogroups were identified among the 56 confirmed cases. *S. flexneri* predominated (58.9%, 33/56), followed by *S. boydii* (23.2%, 13/56), *S. sonnei* (14.3%, 8/56), and *S. dysenteriae* (3.6%, 2/56) (Fig 4). Children aged 12–23 months showed the highest incidence rate of *Shigella* infections. The distribution patterns by age and gender are detailed in Fig 4.

### Clinical characteristics of diarrhea episodes

We recorded 236 diarrheal episodes, with complete data available for 230 cases (Table 4). Of these, 18 (7.8%, 95% CI: 4.7-12.1%) were *Shigella*-positive. Mucoid stools were present in 64.8% of cases (149/230), while bloody stools, a classic feature of shigellosis, occurred in 9.1% (21/230).

**Table 4:** Clinical characteristics of diarrheal cases according to *Shigella* positivity (N = 230).

Characteristics	*Shigella* - (N = 212)	*Shigella*+ (n = 18)	p-value
Gender			
Female	102 (48.1%)	8 (44.4%)	0.765
Age groups (Months)			
0-11	63 (29.7%)	5 (27.8%)	0.768*
12-23	68 (32.1%)	8 (44.4%)	
24-35	47 (22.2%)	2 (11.1%)	
36-47	21 (9.9%)	2 (11.1%)	
48-59	13 (6.1%)	1 (5.6%)	
Type of Diarrhea			
Bloody	17 (9.1%)	4 (22.2%)	0.067*
Mucoid	135 (63.7%)	14 (77.8%)	0.307*
Clinical Features			
Fever	50 (23.6%)	7 (38.9%)	0.149
Vomiting	54 (25.5%)	4 (22.2%)	1.000*
Diarrhea severity (Vesikari score)			
Mild	5 (2.4%)	–	0.757*
Moderate	105 (49.5%)	8 (44.4%)	
Severe	102 (48.1%)	10 (55.6%)	
Nutritional Status			
Stunted (HAZ < -2)	49 (23.1%)	1 (5.6%)	0.132*
Wasted (WHZ < -2)	18 (8.5%)	2 (11.1%)	0.661*
Underweight (WAZ < -2)	33 (15.6%)	1 (5.6%)	0.485*

* Fisher’s exact test used for small cell counts. N = 230 represents diarrheal episodes with complete clinical data; 6 episodes with missing data were excluded.

Clinical comparison between *Shigella*-positive and *Shigella*-negative episodes showed that bloody stools were more frequent in the *Shigella* group (22.2% vs. 8.0%, p = 0.067). Fever accompanied 38.9% of *Shigella*-positive episodes compared to 23.6% of *Shigella*-negative cases (p = 0.149). Neither vomiting (22.2% in *Shigella*+ vs. 25.5% in *Shigella*-, p = 1.000) nor mucoid stools (77.8% in *Shigella*+ vs. 63.7% in *Shigella*-, p = 0.307) significantly predicted *Shigella* infection.

Regarding nutritional status, *Shigella* detection was lower in stunted children (5.6%) compared to non-stunted children (23.1%), though this difference was not statistically significant (p = 0.132). Wasting (11.1% vs 8.5%, p = 0.661) and underweight status (5.6% vs 15.6%, p = 0.485) also showed no significant associations with *Shigella* positivity. Demographic analysis revealed similar positivity by gender (Female: 44.4% in *Shigella*+ vs 48.1% in *Shigella*-, p = 0.765). The highest proportion of *Shigella*-positive diarrheal episodes was observed in the 12–23-month age group (44.4%).

## Discussion

This one-year, community-based cohort study provides important insights into the burden and epidemiology of *Shigella* infection among children under five years living in a peri-urban area of Ouagadougou. The overall *Shigella* incidence of 6.8 cases per 1000 child-months confirms its endemicity in this setting, with particularly high rate in diarrheal cases (39.2/1000 child-months). The predominance of *S. flexneri* (58.9%) aligns with African surveillance data, though our lower *S. dysenteriae* prevalence (3.6%) suggests possible regional variations in serotype distribution [5].

The *Shigella* incidence observed in this study was lower than rates previously reported in sub-Saharan Africa, such as 210.6 episodes per 1,000 child-years (95% CI: 117.0–355.1) described by Khalil et al. in the Global Burden of Disease Study (2018) [5]. This discrepancy is likely attributable to differences in diagnostic methods: while the referenced study used molecular diagnostics, our study relied on stool culture, which is known to be less sensitive [31]. Stool culture’s limitations-including reduced sensitivity due to bacterial viability, sample transport conditions, and selective culture media-likely contributed to underestimation of the true burden [32,33]. Molecular diagnostics, such as quantitative PCR or RLDT (Rapid diagnostic tests and loop-mediated isothermal amplification method), have demonstrated significantly greater sensitivity, detecting up to twice as many cases as stool culture [26]. Based on the adjustment multiplier proposed by McQuade et al. (2020) [33], the true incidence is estimated to be as high as 74.8 cases per 1,000 child-months.

The timing of the study during the peak of the COVID-19 pandemic may have further influenced the findings, as fear of exposure likely reduced health-seeking behavior and contributed to underreporting of diarrheal illnesses. Similar declines in shigellosis incidence were observed in Israel during the pandemic period [34].

Age-specific incidence patterns revealed the highest burden in children aged 12–23 months (aHR = 2.68; 95% CI: 1.11, 6.47; p = 0.033) and 36–47 months (aHR = 2.81; 95% CI: 1.08, 7.33; p = 0.040). This age-dependent pattern, where risk peaks during the weaning period and the transition to independent mobility, is highly consistent with global findings from the Global Enteric Multicenter Study (GEMS) [35]. Regarding the consequences of infection, asymptomatic carriage is associated with adverse outcomes such as stunting and malnutrition [7–9]. Furthermore, *Shigella* has been linked to cognitive deficits in early childhood, as documented by Khalil et al. [6], though it should be noted that Nasrin et al. [9] focused primarily on growth faltering rather than psychomotor development.

While *S. flexneri* was the most prevalent species (58.9%), we observed a higher proportion of *S. boydii* (23.2%) compared to *S. sonnei* (14.3%). This high prevalence of *S. boydii* is notable, as current quadrivalent conjugate vaccines in development primarily target *S. sonnei* and the most common *S. flexneri* serotypes [36]. The regional prominence of *S. boydii* in Ouagadougou highlights the necessity for continued serotype surveillance to ensure that future vaccine formulations provide adequate coverage for West African populations.

The strong association between diarrhea and *Shigella* detection (HR = 13.52; 95% CI: 7.18, 25.45; p < 0.001; multivariate aHR = 15.11; 95% CI: 7.51, 30.42; p < 0.001) reinforces *Shigella*’s role as a major diarrheal pathogen. However, the predominance of mucoid (64.8%) over bloody stools (9.1%) challenges traditional dysentery-focused case definitions, suggesting that current protocols may miss many infections. This has important implications for clinical management and surveillance paradigms [37].

Our multivariate analysis identified key environmental and demographic drivers of *Shigella* risk. Notably, access to public water sources provided a significant protective effect (aHR: 0.52, 95% CI: 0.29–0.94, p = 0.031). This underscores that even in peri-urban settings, the provision of treated communal water can halve the risk of infection compared to private or unimproved sources. Conversely, we observed that children in “Fair” socioeconomic households had a higher hazard of infection than those in “Poor” households (aHR: 2.28; 95% CI: 1.11, 4.68; p = 0.025). This counterintuitive finding may reflect increased social mobility or different community childcare patterns in slightly higher-income households that facilitate exposure, a phenomenon that requires further socio-behavioral investigation.

Several limitations should be considered. First, the focus on a peri-urban area may limit generalizability to rural or urban populations with different socio-environmental conditions [11]. Second, the reliance on stool culture likely underestimated shigelloses incidence compared to more sensitive molecular diagnostics [26,33,35,38]. Third, severe cases requiring hospitalization were not captured, as the study was limited to outpatient settings, potentially missing the full spectrum of disease severity [39]. Fourth, the one-year duration may not be sufficient to capture seasonal or interannual variation in *Shigella* incidence [40]. Additionally, risk factor assessment relied on self-reported data, which is susceptible to recall and social desirability biases, and some variables were only measured at a single time point, which may possibly change over time.

Despite these limitations, the study has notable given interesting inside knowledge. The cohort design enabled collection of both symptomatic and asymptomatic samples, providing a comprehensive estimate of incidence. The combination of active community and passive facility-based surveillance improved case detection, particularly for asymptomatic carriers, and strengthened the reliability of the findings [41]. Although less sensitive than molecular methods, stool culture remains valuable for isolating viable pathogens and conducting antimicrobial resistance profiling, which is essential for guiding treatment and informing public health interventions against antimicrobial resistance [42].

In summary, this study highlights the substantial burden of shigellosis among young children in peri-urban of Ouagadougou and underscores the need for improved diagnostics, targeted interventions, and continued surveillance to inform effective prevention and control strategies.

## Conclusion

*Shigella* remains an important cause of moderate to severe diarrhea in children under five years in Burkina Faso, confirming its endemicity and public health importance in this setting. Our findings demonstrate an overall incidence rate of 6.8 (95% CI: 5.3, 8.9) per 1000 child-months. Among the 56 confirmed incident cases, all four *Shigella* serogroups were identified, with a clear predominance of *S. flexneri* (58.9%) and a notably, high prevalence of *S. boydii* (23.2%).

Multivariate analysis revealed that children aged 12–23 months are at the highest risk of *Shigella* infection, while access to public water sources acts as a significant protective factor, reducing the hazard of infection by 50%. These findings underscore the urgent need to prioritize the provision of safe communal water and targeted hygiene interventions for toddlers during the weaning period.

Surveillance should be improved through the adoption of more sensitive diagnostic methods to better assess the true burden of disease, as conventional stool culture truly underestimates incidence. Furthermore, the regional prominence of *S. boydii* suggests that future multivalent vaccine programs must consider local serogroup diversity to ensure effective coverage. Ultimately, expanding access to sensitive diagnostic tools and characterization of circulating strains are essential to reduce *Shigella*-related morbidity and mortality among young children in Burkina Faso.
