# Supplementary material for: Burden of Shigella infection in children under five years living in a peri-urban area of Ouagadougou, Burkina Faso: A community-based cohort study
Source: PLoS Negl Trop Dis. 2026 Aug 14;20(8):e0014624. doi: 10.1371/journal.pntd.0014624 (PMC13475976; doi:10.1371/journal.pntd.0014624)
Supplement: Supplementary file 1 [file pntd.0014624.s001.docx]

**General comments**

**PNTD-D-25-00346**

This manuscript highlighted the burden of *Shigella* infection in peri-urban area Ouagadougou, Burkina Faso among children less than 5 years

The paper has described the result section well. However, there is limitation in background and methods section. The major drawback of this paper are that 1) the references and text are not matching or the references have not been cited properly, and 2) the recruitment and selection procedures of study are not clear or not described in methods section.

Overall recommendation: Re-write and re-submit the manuscript

**The following comments are to review and address/update in the manuscript:**

1. Page 2, L 17-18: The duration of 12 months longitudinal study and study time period (Dec 2020-Mar 2022). The time period is also not matching with page 7, line 128. Please check and update.
2. P-4. L 60: Ref # 1 did not analyze global burden and death. Provide appropriate mortality data and reference.
3. P-4, line 63: The reference # 2 is not appropriate. This paper (ref#) represented data from sub-Saharan Africa.
4. P-4, Line 69-70: Give a reference
5. P-4, Line 72: Ref # 4 does not reflect global data
6. P-4, Line 73: Cite appropriate reference and the text
7. Line 76-77 (Ref 6, 3, 7): provide appropriate citations.
8. Page 5, L 99: Find appropriate reference.
9. Page 7, L 128: Check the time period with abstract and make consistent
10. Page 7 (Study design and participants): The enrolment procedures of study participants are not clear. What were the criteria to be part of the study and to be excluded from the study? What was the population census/demographic characteristics in the study area? Where was the child enrolled? I see screening (page 9, line 158) was done at the health centre.
11. Page 8, Line: Explain more details about parent education
12. Page 8, line-145-158: Definitions are not the part of laboratory method
13. Page 8, line 149-150: How was “after a period” without diarrhea or dysentery defined?
14. Page 8, line 148: The ref # 25 is not WHO guidelines
15. Page 8, line 156: Is it standard reference for moderate-to-severe diarrhea?
16. Page 9, Stool Samples collection: How stool samples were collected in health facility from asymptomatic cases? There are also concern to collect stool samples from diarrhea cases
17. Page 169, ref 27: Is the ref # 27 appropriate?
18. Page 10-11, L 198-201: Give reference
19. Page 11: How total child-month at risk was calculated for asymptomatic cases or who did not attend in sentinel health centres after an episode of diarrhea?
20. Page 11, L-219: ref # 28 is not WHO growth standards.
21. Page 13, Table 1: Make number of numerical value consistent after decimal point.
22. Page 13, Table 1: A trained research team collected data. However, missing values for mother’s age, education, occupation, Marital status, Mother’s number of children, socioeconomic status, main source of drinking water, handwashing habit and type of toilet were high.

Total enrolled: 750 children. However, total number for mother age: 214+536+71=821

23. Page 16, Figure 1. Elaborate for “M”. How will the investigators explain for 750 children present in “M0” visit? How were they invited to participate in the study?
24. Page 16, L 256-257: Please explain clearly incidence by diarrhea severity. The incidence are not matching with table 2 for moderate and severe cases by Vesikari score
25. Page 19 & 21, line 271, table 4: What were the reference value for toilet type
26. Page 24, line 323-324: How the percentage of vomiting (3.4%) and percentage of mucoid stool (8.1%) were calculated?
27. Page 26, L 343: Ref # 29 is not related with culture, molecular diagnostics and sensitivity
28. Page 26-27: check and update appropriate ref.
