# Supplementary material for: Burden of Shigella infection in children under five years living in a peri-urban area of Ouagadougou, Burkina Faso: A community-based cohort study
Source: PLoS Negl Trop Dis. 2026 Aug 14;20(8):e0014624. doi: 10.1371/journal.pntd.0014624 (PMC13475976; doi:10.1371/journal.pntd.0014624)
Supplement: Supplementary file 2 [file pntd.0014624.s003.docx]

**Title:** Burden of *Shigella* infection in children under five living in peri-urban area of Ouagadougou, Burkina Faso: A community-based cohort study.

**Authors and Affiliations:**

Alimatou Héma^1,3*^, Jean Sawadogo^1^, Ben Idriss Soulama^1^, Denise Hien^1^, Amidou Diarra^1^, Samuel S Sermé^1^, Amidou Z Ouédraogo^1^, Issa Nébié^1^, Sophie Houard^2^, Alphonse Ouedraogo^1^, Alfred B Tiono^1^, Mahamoudou Sanou^3¥^, Sodiomon B Sirima^1¥^

^1^ Groupe de Recherche Action en Santé (GRAS), Ouagadougou, Burkina Faso ;

^2^ European Vaccine Initiative; Heidelberg, Germany

^3^ Université Joseph KI-ZERBO (UJKZ), Ouagadougou, Burkina Faso;

^*^ Corresponding author: [a.hema@gras.bf](mailto:a.hema@gras.bf)	(HA)

^¥^ These authors contributed equally to this work.

**Abstract**

A clear understanding of *Shigella* epidemiology and associated risk factors is essential for developing effective vaccines and control strategies against this leading cause of childhood diarrheal disease. We conducted a 12-month longitudinal cohort study in a peri-urban area of Ouagadougou, Burkina Faso, enrolling 750 children under five years of age from December 2020 to March 2021. Each child was followed for 12 months, with the final follow-up concluding in March 2022. Active and passive surveillance were employed to capture symptomatic and asymptomatic infections. Stool samples (n=2,401), including 236 from diarrheal episodes, were analyzed using conventional microbiological methods with species confirmation by the BD Phoenix M50 system. Data were analyzed using STATA/MP 17.1.

*Shigella* was isolated from 56 incident cases, yielding an incidence rate of 6.8 cases per 1,000 child-months (95% CI: 5.3–8.9). *Shigella flexneri* predominated (58.9%), followed by S. boydii (23.2%), S. sonnei (14.3%), and S. dysenteriae (3.6%). The incidence rate of *Shigella* among diarrheal cases was 38.8 (95% CI: 24.4, 61.5) with a substantial carriage rate (4.9/1,000 child-months). Clinically, shigellosis was strongly associated with diarrhea, predominantly presenting as mucoid stools, with a smaller proportion exhibiting bloody diarrhea. Risk factor analysis identified young age (particularly 12–23 months). Unexpectedly, reported regular handwashing was associated with higher detection rates, possibly reflecting contamination of water used for hygiene or behavioral factors.

This study quantified a *Shigella* incidence of 6.8 cases per 1,000 child-months among children under five in peri-urban Burkina Faso, with *Shigella flexneri* (58.9%) as the predominant serotype. The substantial incidence among diarrheal cases and notable asymptomatic carriage underscore the public health burden. These findings provide foundational data on *Shigella* prevalence and serotype distribution critical for guiding future vaccine development and targeted public health interventions in the region. Importantly, the results highlight the need to strengthen WASH programs, improve diagnostic capacity, and enhance antimicrobial resistance surveillance to mitigate transmission and impact among young children.

**Author Summary**

*Shigella* remains a major threat to children in resource-limited settings, causing substantial diarrheal morbidity. Our year-long study in Burkina Faso tracked 750 under-five children through community- and facility-based surveillance, analyzing 2,401 stool samples. We found an overall incidence of 6.8 culture-confirmed *Shigella* cases/1,000 child-months. 7.8% of diarrheal episodes were *Shigella*-positive, predominantly with mucoid (64.8%) rather than bloody stools (9.1%). *S. flexneri* caused nearly 60% of infections. Culture-based methods likely underestimated true burden. These results highlight the need for enhanced diagnostics to capture *Shigella* cases; vaccines effective against predominant local serotypes; context-specific prevention strategies accounting for non-dysenteric presentations. The study provides critical baseline data for upcoming vaccine trials while revealing important gaps in current surveillance paradigms.

**Introduction**

Acute diarrheal diseases represent a significant global public health challenge, causing an estimated 1.17 million deaths annually worldwide, with the highest mortality burden observed among children under five years of age, especially in low-income countries. The World Health Organization (WHO) identifies diarrheal disease as a significant health threat, responsible for a substantial proportion of deaths among children under five years of age, particularly in low- and lower-middle-income countries. Sub-Saharan Africa and South Asia bear the greatest burden of these deaths, reflecting ongoing disparities in sanitation, nutrition, and healthcare access worldwide [1].

*Shigella* is the second most common pathogen responsible for infectious diarrhea. Diarrhea caused by *Shigella*, known as shigellosis, is an acute invasive enteric infection manifested mainly by bloody diarrhea. *Shigella* belongs to the *Enterobacterales* family and comprises four distinct serogroups: *Shigella dysenteriae, Shigella flexneri*, *Shigella boydii* and *Shigella sonnei*. The geographical distribution of these species varies according to socio-economic level [2]. *Shigella flexneri* predominates in developing countries, while *Shigella sonnei* is more common in developed countries [2,3]. On the other hand, although *Shigella dysenteriae* and *Shigella boydii* are more often found in developing countries, globally they are more rarely isolated compared to *Shigella flexneri* [4].

Shigellosis is one of the most prevalent diarrheal diseases in developing countries [3]. It contributes to substantial morbidity, with an estimated over 267 million cases annually, nearly 69% of which occur in children under five, particularly in developing countries, with approximately 64,000 deaths in children under five each year [5]. In these children, shigellosis can also impair psychomotor development [6], even in asymptomatic carriers [7–9]. The persistence of *Shigella* after diarrheal episodes often leads to asymptomatic carrier states, complicating efforts to reduce shigellosis [9]. Numerous studies have documented a high number of asymptomatic *Shigella* carriers in developing countries, perpetuating the cycle of infection [8,9].

In endemic areas, the lack of adequate latrines, poor hygiene, and limited access to clean drinking water contribute to the spread of *Shigella* [10]. Climatic factors and unfavorable socio-economic conditions, particularly in sub-Saharan Africa's tropical climate, further facilitate its transmission [11]. Given these challenges, vaccination remains the most effective means of prevention [12–14]. However, comprehensive data on *Shigella* epidemiology are essential for designing effective vaccines and guiding vaccination policies [15].

The deficiency in technical capacity for surveillance in many developing countries, including Burkina Faso, contributes to a substantial underestimation of the burden of shigellosis [5]. Moreover, limited data on shigellosis in community settings, with a predominant focus on hospital studies [10,16,17], further hinder an accurate representation of the true burden and diversity of *Shigella*.

The management of shigellosis is further complicated by the increasing threat of antimicrobial resistance (AMR) due mainly to misuse, overuse or abuse of antibiotics [18]. This misuse of antibiotics accelerates the development of drug-resistant pathogens, making infections harder to treat and control. AMR in *Shigella* has become a significant concern, as resistant strains are associated with higher rates of treatment failure, prolonged illness, and increased mortality. The WHO has prioritized *Shigella* as a critical target for new antimicrobial therapies and interventions due to this growing resistance [19,20].

In developing countries, the misuse of antibiotics is often driven by factors such as inadequate healthcare infrastructure, lack of regulation, and easy over-the-counter access to antibiotics without prescriptions [21]. Additionally, the overuse of antibiotics in agriculture and animal husbandry contributes to the development and spread of resistant strains. As a result, the treatment options for shigellosis are becoming increasingly limited, posing a severe public health threat [22].

Enhanced surveillance capacity improves the detection and estimation of shigellosis, leading to more appropriate vaccine policies and antimicrobial stewardship programs.

This study is one of the objectives of the ShigaPlexIM consortium, which aims to characterize the epidemiology of *Shigella* infections across multiple African sites. As a component of this effort, we conducted a longitudinal baseline epidemiological study in peri-urban areas of Ouagadougou, Burkina Faso, to estimate the incidence of *Shigella* infections (symptomatic and asymptomatic) among children under five years of age. Secondary objectives included characterizing the circulating *Shigella* serogroups and identifying key risk factors associated with the burden of infection within this population.

**Methods**

**Study site**

The study was conducted in a peri-urban community on the outskirts of Ouagadougou, Burkina Faso’s capital city. Located in the Sahel region, Burkina Faso covers a total area of 274,200 km^2^. Ouagadougou is located in the central region of the country, which is characterized by the highest population growth rate at 4.42% [23]. The specific study area included the neighborhoods of Polesgo and Nioko 2, both unplanned growth zones situated near the Kossodo industrial zone [24]. These neighborhoods fall within the Kossodo health district.

The surroundings of Ouagadougou, akin to many outskirts of African capital cities, consist of informal settlements where the most vulnerable populations reside [25]. The urban planning and environmental services in these peri-urban areas are inadequate, marked by high housing density, poor road networks, a lack of access to clean drinking water, and insufficient sanitation measures. As of June 2010, these two areas are made of approximately 37,878 households [24]. The challenging living conditions in these peri-urban areas underscore the importance of studying the incidence of *Shigella*-associated diarrheal diseases to inform public health interventions and improve the well-being of the communities. Figure 1 provides a detailed map of the peri-urban study area, showing the geographical locations of the Polesgo and Nioko 2 neighborhoods, key landmarks, and the health facilities involved in participant recruitment.

**Fig 1. Map of Ouagadougou city showing the study sites with neighborhood boundaries (left), the map of Africa and the map of Burkina Faso (right)**

**Study design and participants**

This research formed part of a multisite, longitudinal cohort study under the ShigaPlexIM consortium, conducted in comparable communities in Burkina Faso and Zambia. The present manuscript presents data from the Burkina Faso site, where 750 children under five years were enrolled between December 2020 and March 2021 and followed for 12 months. The overall study period spanned 16 months (December 2020 to March 2022), reflecting the enrollment phase followed by individual 12-month follow-up for each child post-enrollment.

The study was conducted in the Nongr-Massom health district of Ouagadougou, encompassing the peri-urban neighborhoods of Polesgo (six quartiers) and Nioko 2 (eight zones). Recruitment was performed through a standardized community-based procedure aligned with the study’s Standard Operating Procedure (SOP). Community Health Workers collaborated closely with local health authorities, community leaders, and residents to facilitate engagement and mobilization.

Households with children under five were identified by Community Health Workers, who informed families about the study objectives, procedures, and participation benefits. Interested parents or guardians registered their children on volunteer lists and received unique identification tickets. To ensure equitable representation, quota targets were set at approximately 300 children from Polesgo and 450 from Nioko 2. When volunteer numbers exceeded quotas, a simple random drawing without replacement was conducted publicly to select participants fairly.

Selected families were then invited to attend screening and enrollment visits at designated health facilities: Polesgo Health and Social Promotion Center (CSPS) for Polesgo residents and Kossodo Medical Center (CMA) for Nioko 2 residents. Screening involved confirmation of eligibility, informed consent procedures, and baseline data collection. Eligible participants were permanent residents without plans to relocate during the subsequent 12-month follow-up. Exclusion criteria included children born after recruitment, known or suspected immunodeficiency, major congenital anomalies, recent immunosuppressive therapy, or concurrent participation in other clinical studies.

After enrollment (designated as Month 0, M0), trained clinical staff conducted scheduled and unscheduled home visits to collect epidemiological, clinical, and laboratory data throughout follow-up. Active surveillance included community sensitization to diarrheal symptoms and encouragement to seek prompt care at sentinel health centers, where stool samples were collected, cases were managed, and clinical data recorded.

The last enrolled participant completed follow-up in March 2022, concluding the longitudinal surveillance period**.**

**Definitions**

Diarrhea was defined as three or more loose stools per 24-hour period according to the official WHO guidelines on diarrhea management [26].

A new diarrheal episode was defined as any occurrence of diarrhea at least seven days after the last diarrhea or dysentery-free period, following WHO recommendations for episode separation. Any stool sample positive for *Shigella* was defined as an episode of *Shigella* infection. A case of asymptomatic *Shigella* infection was defined as any non-diarrheal stool sample that tests positive for *Shigella* by stool culture. *Shigella* cases were confirmed by phenotyping, including BD Phoenix M50.

Moderate to Severe Diarrhea (MSD) is defined as a diarrheal illness lasting less than one week in a child, characterized by at least three loose stools within 24 hours accompanied by two or more of the following clinical signs: lethargy, sunken eyes, poor or eager drinking, slow skin pinch (skin turgor), or presence of dysentery [27,28].

Asymptomatic cases were defined as children seen at the health center without diarrhea during a planned visit (at screening, month 6 and month 12 visits).

**Sample size calculation**

Sample size estimations accounted for the multisite design of the ShigaPlexIM baseline study, which was conducted in Burkina Faso and Zambia. To estimate the incidence of *Shigella* moderate-to-severe diarrhea (MSD) with sufficient power and precision, detecting a minimum of 22 events required a cohort size of 734 children. This sample size yielded a two-sided 95% confidence interval with a precision width of 0.025, assuming a hazard rate (λ) of 0.03 and 97% censoring. Considering an anticipated 2% loss to follow-up, the minimum cohort size was adjusted to 750 children. Accordingly, to ensure adequate statistical power at each site, we planned to recruit 750 children per site in Burkina Faso and Zambia.

**Laboratory Methods**

**Stool samples collection**

Stool samples were collected at the “Centre Medical avec Antenne chirurgicale (CMA)” of Kossodo and the “Centre de Santé et de Promotion Sociale (CSPS)” of Polesgo. For asymptomatic children attending scheduled visits, parents or guardians were provided with sterile containers and instructed by trained study staff to collect stool samples either at home or at the health facility before the visit. For children presenting with diarrhea, trained healthcare personnel collected fresh stool samples directly at the health facility to ensure prompt and quality specimen collection. All stool samples were placed in Cary-Blair transport medium and transported to the Groupe de Recherche Action en Santé (GRAS) bacteriology-virology laboratory at a controlled temperature between 2 and 8°C for microbiological analysis.

**Culture and identification of *Shigella* isolates**

Stool samples were inoculated on MacConkey (MA) and Xylose Lysine Deoxycholate (XLD) agar and selenite F broth (Liofilchem®, Roseto degli Abruzzi, Italy). Inoculated plates were incubated at 35–37°C and examined after 18–24 hours according to conventional stool culture techniques [29]. Subcultures from selenite F broth were performed on Salmonella-*Shigella* agar. Non-lactose fermenting colonies were identified through standard biochemical tests. *Shigella* suspected colonies (small pale colonies on MacConkey and red colonies on XLD) were stabbed in Triple Sugar Iron Agar (TSI), Sulphur Indole Motility Agar (SIM) and Lysine Decarboxylase Citrate Agar (LDC) (Beckton & Dickinson, NJ, USA), and subjected to the urease production test. After these tests, API 20E system biochemical tests (BioMérieux, UK). *Shigella* serogroups were identified using polyvalent antisera (Bio-Rad, France). *Shigella flexneri* sv 2b Gp B ATCC 12022 and *Shigella sonnei* ATCC 25931 served as the controls.

Double confirmation of the *Shigella* diagnosis was performed using the BD Phoenix M50 identification Automated Microbiology system (BD Diagnostic Systems version 2.20.0.0/V6,81A(x-US). The Phoenix identification method uses modified conventional, fluorogenic, and chromogenic substrates. Combination panels NMIC/ID-435 (catalogue no. 449044) were used for both identification and susceptibility testing. The ID side contains 45 wells with dried biochemical substrates and 2 fluorescent control wells. The ID broth was inoculated with bacterial colonies adjusted to a 0.5 McFarland standard by using a CrystalSpec nephelometer (BD Diagnostics), according to the manufacturer's recommendations. The suspension was then poured into the ID side of the Phoenix panel after an aliquot (25 μl) was removed for antimicrobial susceptibility testing (AST). The specimen was logged and loaded into the instrument within the specified timeline of 30 minutes. Quality control and maintenance were performed according to the manufacturer's recommendations. The results were analyzed using Epicenter data management software version 3.01A (BD Diagnostic Systems) after 16 hours of incubation.

**Statistical analysis**

Data were analyzed using Stata software version 17 MP (StataCorp LLC). The burden of *Shigella*-attributable moderate-to-severe diarrhea (MSD) and associated risk factors were estimated using survival analysis techniques.

Categorical variables (e.g., maternal education, water source) were recoded into binary or grouped categories to facilitate analysis. String variables were encoded appropriately for regression modeling. Children’s nutritional status at enrollment was assessed using weight and length measurements with weight-for-age (WAZ), height-for-age (HAZ), and weight-for-height (WHZ) Z-scores were computed according to WHO child growth standards. Undernutrition was defined as WAZ < –2 (underweight); HAZ < –2 (stunted); WHZ < -2 (wasted).

A descriptive analysis summarized baseline characteristics using counts and proportions for categorical variables and means ± standard deviations (SD) for continuous variables. High numbers of missing values for some maternal and household variables were noted; these are addressed with explanations in the Methods and footnotes to Table 1.

The primary outcome was the time to the first episode of *Shigella*, defined by stool culture positivity. Time-at-risk was calculated in child-months for all participants, including those asymptomatic or not attending sentinel health centers, by summing individual exposure periods until infection or censoring. Incidence rates were computed as the number of first-episode cases divided by the total child-months at risk, expressed per 1,000 child-months.

Predictors of time-to-first *Shigella* infection were assessed using Cox proportional hazards regression. Univariable (bivariate) models were first applied for initial screening to identify potential risk factors associated with *Shigella* infection. Subsequently, all relevant explanatory variables were entered into multivariable models to estimate adjusted hazard ratios (HRs), controlling for confounding factors. For time-varying covariates such as diarrhea episodes, extended Cox models were used.

Variable selection in multivariable modeling employed backward stepwise elimination, retaining predictors with p < 0.05. Hazard ratios were reported with 95% confidence intervals (CIs). The proportional hazards assumption was checked using Schoenfeld residuals and time-interaction terms, confirming model validity.

Covariates in the analysis included sociodemographic factors (maternal education, occupation), household-level factors (toilet type, water source, waste disposal), and child nutritional status indicators based on weight and length measurements. Weight-for-age (WAZ), height-for-age (HAZ), and weight-for-height (WHZ) Z-scores were calculated according to the World Health Organization (WHO) child growth standards (2006). Undernutrition was defined using standard cutoffs: WAZ < - 2 indicating underweight, HAZ < - 2 indicating stunting, and WHZ < - 2 indicating wasting [30].

**Ethical approval**

The study was approved by the Health Research Ethics Committee (HREC) of Burkina Faso, with the project reference number: 2020-3-055 dated 25 March 2020, Protocol Version: 1.0, February 2020 ShigOraVax (NCT04312906). Written informed consent was obtained from the children's parents or guardians at the time of enrolment for participation in the study and storage of samples for future use. The anonymity and confidentiality of the information collected were preserved by assigning identification numbers, so as not to infringe on the dignity of the participants. Information provided by participants was kept confidential and password protected.

**Results**

**Study Population Characteristics**

We enrolled 750 under-five children (51.6% male) in this 12-month study. Participants had a median age of 28.1 months (IQR: 15.0; range: 2-60) with right-skewed distribution (skewness=22.2). Anthropometric measures showed mean weight 11.1±2.8 kg (median: 12.0, IQR: 4.2) and height 89.8±37.3 cm (median: 88.8, IQR: 17). Nutritional deficiencies included stunting (26.3%, HAZ<-2), wasting (5.6%, WHZ<-2), and underweight (12.3%, WAZ<-2). Most mothers were >25 y ears (71.5%), married (94.4%), with 50.1% having no formal education and 49.3% unemployed. The characteristics of the study participants and their households are described in Table 1.

**Table 1.** Baseline characteristics of the study participants

| **Characteristics** | **Frequency n (%)** |
| --- | --- |
| Age categories (months) (n=750) |  |
| 0-11 | 124 (16.5) |
| 12-23 | 207 (27.6) |
| 24-35 | 181 (24.1) |
| 36-47 | 135 (18.0) |
| 48-59 | 103 (13.7) |
| Gender (n=750) |  |
| Male | 387 (51.6) |
| Female | 363 (48.4) |
| Mean weight at enrolment (Kg) | 11.1 ± 2.8 |
| Missing | 4 |
| Nutritional status at enrolment (n=745) |  |
| Underweighted (WAZ < -2.0) | 92 (12.3) |
| Stunted (HAZ< -2.0) | 197(26.3) |
| Wasted (WHZ < -2.0) | 42 (5.6) |
| Normal (WAZ, HAZ/ WHZ ≥ -2.0) | 415 (55.6) |
| Mothers’ characteristics |  |
| Mother's Age (n=750) |  |
| 25 years and younger (≤25 years) | 214 (28.5) |
| Over 25 years (>25 years) | 536 (71.5) |
| Mother formal education (n=671)* |  |
| No formal education | 336 (50.1) |
| Some formal education | 335 (49.9) |
| Mother's occupation (n=690)* |  |
| Housewife | 340 (49.3) |
| Income generating activity (informal/formal sector) | 350 (50.7) |
| Mother’s marital status (n=690)* |  |
| Single | 39 (5.7) |
| Married | 651 (94.3) |
| Mother's number of children (n=690)* |  |
| Less than 3 living children | 409 (59.3) |
| 3 or more living children | 281 (40.7) |
| **Household level factors** |  |
| Socioeconomic status of the household (n=690) |  |
| Poor | 250 (36.2) |
| Middle | 211 (30.6) |
| Rich | 229 (33.2) |
| Water, sanitation and hygiene (WASH) of the household |  |
| Main source of drinking water (n=690)* |  |
| Piped into house | 182 (26.4) |
| Public tap/Borehole | 508 (73.6) |
| Handwashing habit (n=689)* |  |
| Regularly wash hand | 381 (55.3) |
| Occasionally | 308 (44.7) |
| Type of toilet into the house (n=690)* |  |
| Improved sanitation | 559 (81.0) |
| Unimproved sanitation | 131 (19.0) |

**Note: The total number of participants is n=750. Denominators less than 750 for maternal and household variables (e.g., Mother's formal education, n=671; Mother's occupation, n=690) reflect missing data due to non-response during the baseline socio-demographic interview. All percentages for these characteristics are calculated based on the number of valid responses for each specific item.*

***Shigella* incidence**

During the twelve-month follow-up period, a total of 2,401 stool samples (236 diarrheal and 2,165 non-diarrheal samples) were tested for *Shigella* by culture as described in the flowchart (Figure 2).

**Fig 2. Study flowchart**

*Legend: This flowchart illustrates participant enrollment and stool sample collection during a twelve-month cohort study in peri-urban Ouagadougou, Burkina Faso. A total of 750 children were enrolled and provided non-diarrheal stool samples at baseline (Month 0), Month 6, and Month 12. Passive case detection identified 236 diarrheal episodes from 181 children during follow-up. In total, 2,401 stool samples (236 diarrheal and 2,165 non-diarrheal) were collected and tested for Shigella using culture methods.*

From all stool samples, 76 cases of *Shigella* were isolated, of which sixteen cases of *Shigella* had occurred, after the first incident episode, and therefore were not included in the analysis. The total of 60 incident cases of confirmed *Shigella* cases resulted and four participants had their *Shigella* episode the day of their enrollment. These 56 incident cases result with an incidence rate of *Shigella* infections of 6.8 episodes (95 % CI: 5.2, 8.8) per 1000 child-months. The incidence rates of *Shigella* infections according to sex were 7.1 (95 % CI: 4.9, 10.3) and 6.5 (95 % CI: 4.5, 9.5) respectively for females and males. According to age groups, the highest incidence of overall *Shigella* infections occurred in children aged between 12-23 months with 10.1 episodes per 1000 child-months. The incidence rate of asymptomatic cases of *Shigella* was 4.9 (95 % CI: 3.6, 6.8) per 1000 child-months and the incidence of diarrheal cases was 38.8 (95 % CI: 24.4, 61.5) per 1000 child-months (Table 2). Of the cases of diarrhea, the incidence estimate by diarrhea severity was 28.1 (95 % CI: 14.0, 56.2) for moderate cases and the incidence of the severe cases was 47.2 (95 % CI: 25.4, 87.7).

**Table 2. Incidence of *Shigella* infection in children under five years of age**

| **Child characteristics** | **Incidence of *Shigella* (95% CI) per 1000 child-months** |
| --- | --- |
| Overall | 6.8 (5.2, 8.8) |
| Type of stools |  |
| Diarrheal | 35.7 (22.5, 56.7) |
| No-diarrheal | 4.9 (3.6, 6.8) |
| Age (months) |  |
| 0-11 | 6.4 (3.3, 12.3) |
| 12-23 | 10.0 (6.6, 15.2) |
| 24-35 | 4.0 (2.0, 8.0) |
| 36-47 | 8.7 (5.1, 15.1) |
| 48-59 | 3.5 (1.3, 9.3) |
| Sex |  |
| Female | 7.1 (4.9, 10.3) |
| Male | 6.5 (4.5, 9.5) |
| Nutritional status at enrollment (z-score) |  |
| Underweight (WAZ < -2.0) |  |
| Yes | 3.0 (1.0, 9.4) |
| No | 7.4 (5.6, 9.6) |
| Stunting (HAZ< -2.0) |  |
| Yes | 4.6 (2.5, 8.6) |
| No | 7.6 (5.7, 10.1) |
| Wasting (WHL < -2.0) |  |
| Yes | 6.5 (2.1, 20.0) |
| No | 6.9 (5.2, 9.0) |
| Diarrheal characteristics |  |
| Diarrheal severity (Vesikari score) |  |
| Mild | - |
| Moderate | 28.1(14.0, 56.2) |
| Severe | 47.2 (25.4, 87.7) |
| Mucus in stools |  |
| Yes | 46.3 (27.4, 78.2) |
| No | 23.2 (8.7, 61.7) |
| Blood in stools |  |
| Yes | 105.1 (39.5, 280.2) |
| No | 30.1 (17.8, 50.8) |
| Fever |  |
| Yes | 58.2 (27.8, 122.2) |
| No | 28.7 (15.9, 51.8) |
| Vomit |  |
| Yes | 35.7 (13.4, 95.1) |
| No | 35.7 (21.2, 60.3) |
| Water, sanitation and hygiene (WASH) |  |
| Household water source |  |
| Piped into house | 9.9 (6.4, 15.3) |
| Public tap /borehole | 6.3 (4.6, 8.8) |
| Regularly wash hand |  |
| Yes | 9.0 (6.5, 12.3) |
| No | 5.2 (3.3, 8.2) |
| Type of toilet into the house |  |
| Yes | 7.6 (5.8, 10.1) |
| No | 5.1(2.3, 11.4) |

**Risk factors associated with *Shigella* infection**

In the univariate analysis, the type of stool sample (diarrheic or non-diarrheic) was identified as the most crucial factor associated with *Shigella* infection. Indeed, having diarrhea showed the strongest association with *Shigella* infection (Hazard ratio: 13.5 [95% CI: 7.2, 25.4]) compared with asymptomatic cases, with strong statistical evidence (p<0.001). Individuals with diarrheic stools were more than thirteen times more likely to have *Shigella* infection compared to those with non-diarrheic stools. This underscores the importance of *Shigella* as a causative agent of diarrhea.

Among household-level WASH variables; using public water sources showed a borderline significant protective effect compared to other sources (HR: 0.6 [95% CI: 0.4-1.1], p=0.076). Having a covered water container showed a non-significant protective trend (HR: 0.7 [95% CI: 0.3-1.7], p=0.404). Improved toilet type showed a non-significant protective trend (HR: 0.6 [95% CI: 0.3-1.5], p=0.270). Regular handwashing showed a non-significant protective trend (HR: 0.7 [95% CI: 0.4-1.2], p=0.150). Open defecation showed no significant association (HR: 1.1 [95% CI: 0.5-2.2], p=0.800)

Participant-level variables showed limited associations: age categories showed no significant differences (p=0.081 for overall test). Sex showed no significant association (HR: 1.1 [95% CI: 0.7-1.9], p=0.682). Stunting showed a non-significant protective trend (HR: 0.6 [95% CI: 0.3-1.2], p=0.181). Wasting showed no significant association (HR: 0.9 [95% CI: 0.3-2.8], p=0.835).

Socioeconomic factors: maternal education (some formal vs none) showed no significant association (HR: 1.5 [95% CI: 0.8-2.5], p=0.183). Household wealth (middle or rich vs poor) showed no significant associations (p=0.679 for overall test)

The analysis confirms that diarrheic stool type is by far the strongest predictor of *Shigella* detection, with no other factors reaching statistical significance in univariate analysis. Several WASH-related factors showed suggestive but non-significant protective trends, including use of public water sources, having covered water containers, improved toilet facilities, and regular handwashing practices.

**Table 3. Risk factors associated with *Shigella* infection in children under five in peri-urban area of Ouagadougou, Burkina Faso.**

| Variable | **Univariate analysis** | **p-value** | **Multivariate analysis** | **p-value** |
| --- | --- | --- | --- | --- |
| **Diarrheal stool (Ref: Non-diarrheal)** | **13.5 (7.2, 25.4)** | **<0.001** | **15.1 (7.5, 30.4)** | **<0.001** |
| **Child characteristics** |  |  |  |  |
| **Age groups (Ref: 0–11 months)** |  | **0.081** |  | **0.035*** |
| 12–23 months | 1.6 (0.7, 3.4) | 0.244 | **2.7 (1.1, 6.5)** | **0.033** |
| 24–35 months | 0.6 (0.2, 1.6) | 0.343 | 1.3 (0.4, 3.6) | 0.660 |
| 36–47 months | 1.4 (0.6, 3.3) | 0.417 | **2.8 (1.1, 7.3)** | **0.040** |
| 48–60 months | 0.6 (0.2, 1.9) | 0.390 | 1.2 (0.3, 4.3) | 0.771 |
| **Female sex (Ref: Male)** | 1.1 (0.7, 1.9) | 0.682 | 1.1 (0.6, 1.9) | 0.815 |
| **Stunted (Ref: Not stunted)** | 0.6 (0.3, 1.2) | 0.181 | 0.6 (0.3, 1.3) | 0.199 |
| **Wasted (Ref: Not wasted)** | 0.9 (0.3, 2.8) | 0.835 | 0.9 (0.3, 3.0) | 0.852 |
| **Mother’s education (Ref: None)** |  |  |  |  |
| Some formal education | 1.5 (0.8, 2.5) | 0.183 | 1.46 (0.8, 2.6) | 0.208 |
| **Household characteristics** |  |  |  |  |
| **Socioeconomic status (Ref: Poor)** |  | **0.679** |  | **0.025** |
| Fair | 1.3 (0.7, 2.4) | 0.446 | **2.3 (1.1, 4.7)** | **0.025** |
| Rich | 1.0 (0.5, 1.9) | 0.977 | 2.1 (1.0, 4.7) | 0.059 |
| **Water, sanitation, and hygiene (WASH)** |  |  |  |  |
| **Main water source (Ref: Other)** |  |  |  |  |
| Public water source | **0.6 (0.4, 1.1)** | **0.076** | **0.5 (0.3, 0.9)** | **0.031** |
| **Regular handwashing (Ref: No)** | 0.7 (0.4, 1.2) | 0.150 | 0.8 (0.4, 1.5) | 0.434 |
| **Toilet type (Ref: Improved** **sanitation)** | 0.6 (0.3, 1.5) | 0.270 | 0.7 (0.3, 1.7) | 0.415 |
| **Covered water container (Ref: No)** | 0.7 (0.3, 1.7) | 0.404 | 0.7 (0.2, 2.1) | 0.492 |
| **Open defecation (Ref: No)** | 1.1 (0.5, 2.2) | 0.800 | 1.0 (0.5, 2.3) | 0.913 |

**Age category overall significance in univariate (p = 0.081) but significant subgroups in multivariate. For the variable "Toilet type", the reference category is "Improved sanitation." Hazard ratios (HR) represent the relative risk of Shigella infection among children living in households with unimproved toilets compared to those with improved sanitation facilities.*

*Proportional hazards assessment*

Visual inspection of log-log survival curves demonstrated parallel trajectories between groups (Figure 3), supporting the proportional hazards assumption for diarrhea status (log-rank test p=0.765). The constant vertical separation of curves indicates the hazard ratio remained stable throughout follow-up, with no evidence of time-dependent effects (Schoenfeld residuals test p=0.812).

**Fig 3.** **Log-log survival curves by diarrhea status.**

Parallel curves suggest proportional hazards are met for diarrhea (0=absent, 1=present). The vertical distance reflects the constant hazard ratio (HR=13.5) over time.

**Distribution of *Shigella* species**

All four *Shigella* serogroups were identified among 56 confirmed cases. S. flexneri predominated (58.9%, 33/56), followed by S. boydii (23.2%, 13/56), S. sonnei (14.3%, 8/56), and S. dysenteriae (3.6%, 2/56) (Figure 3). Children aged 12-23 months showed the highest incidence of *Shigella* infections. The distribution patterns by age and sex are detailed in Figure 4.

**Fig 4. *Shigella* serogroups distribution according to age groups and sex**

**Clinical characteristics of *Shigella* diarrhea**

We recorded 236 diarrheal episodes, with complete data available for 230 cases. Of these, 18 (7.8%, 95% CI: 4.7-12.1%) were *Shigella*-positive. Mucoid stools were present in 64.8% of cases (149/230), while bloody stools, a classic feature of shigellosis, occurred in 9.1% (21/230). Bloody stools showed higher *Shigella* positivity rate (19.1% vs 6.7% in bloody stools, p=0.0067). Fever accompanied 24.8% of episodes (57/230), with 12.3% (7/57) of febrile cases being *Shigella*-positive (p=0.149). Neither vomiting (6.9% in vomiting cases vs. 8.1% in non-vomiting cases, p=1.000) nor mucoid stools (9.4% in mucoid cases vs. 4.9% in non-mucoid cases, p=0.307) significantly predicted *Shigella* infection. Stunted children had a low *Shigella* detection rate (2.0%), compared to 9.4% in non-stunted children, though this difference was not significant (p=0.132). Wasting (10.0% vs 7.6%, p=0.661) and underweight status (2.9% vs 8.7%, p=0.485) showed no significant associations. Demographic analysis revealed similar positivity by gender (male: 8.3% vs female: 7.3%, p=0.765) and highest detection in 12-23-month-olds (10.5%).

**Table 4.** Clinical characteristics of MSD cases with *Shigella* positivity

| **Characteristics** | **Total (N=230)** | ***Shigella*+ (n=18, 7.8%)** | **p-value** |
| --- | --- | --- | --- |
| **Gender** |  |  |  |
| Male | 120 (52.2%) | 10 (8.3%) | 0.765 |
| Female | 110 (47.8%) | 8 (7.3%) |  |
| **Age groups (Months)** |  |  |  |
| 0-11 | 68 (29.6%) | 5 (7.4%) | 0.768* |
| 12-23 | 76 (33.0%) | 8 (10.5%) |  |
| 24-35 | 49 (21.3%) | 2 (4.1%) |  |
| 36-47 | 23 (10.0%) | 2 (8.7%) |  |
| 48-60 | 14 (6.1%) | 1 (7.1%) |  |
| **Type of Diarrhea** |  |  |  |
| Bloody | 21 (9.1%) | 4 (19.1%) | 0.067* |
| Without bloody stools | 209 (90.9%) | 14 (6.7%) |  |
| Mucoid | 149 (64.8%) | 14 (9.4%) | 0.307* |
| Without mucoid stools | 81 (35.2%) | 4 (4.9%) |  |
| **Clinical Features** |  |  |  |
| Fever | 57 (24.8%) | 7 (12.3%) | 0.149 |
| Without fever | 176 (75.5) | 11 (6.4%) |  |
| Vomiting | 58 (25.2%) | 4 (6.9%) | 1.000* |
| Without vomiting | 172 (74.8%) | 14 (8.1%) |  |
| **Diarrhea severity (Vesikari score)** |  |  |  |
| Mild | 5 (2.2%) | - | 0.757* |
| Moderate | 113 (49.1%) | 8 (7.1%) |  |
| Severe | 112 (48.7%) | 10 (8.9%) |  |
| **Nutritional Status** |  |  |  |
| Stunted (HAZ < -2) | 50 (21.7%) | 1 (2.0%) | 0.132* |
| Non-stunted | 180 (78.3%) | 17 (9.4%) |  |
| Wasted (WHZ < -2) | 20 (8.7%) | 2 (10.0%) | 0.661* |
| Non-wasted | 210 (91.3%) | 16 (7.6%) |  |
| Underweight (WAZ < -2) | 34 (14.8%) | 1 (2.9%) | 0.485* |
| Non-underweight | 196 (85.2%) | 17 (8.7%) |  |

* Fisher's exact test used due to small cell counts (expected frequency < 5). All other p-values were derived from the Pearson Chi-squared test.

**Discussion**

This one-year, community-based cohort study provides important insights into the burden and epidemiology of *Shigella* infection among children under five living in a peri-urban area of Ouagadougou. The overall *Shigella* incidence of 6.8 cases per 1000 child-months confirms its endemicity in this setting, with particularly high rates in diarrheal cases (38.8/1000 child-months). The predominance of S. flexneri (58.9%) aligns with African surveillance data, though our lower S. dysenteriae prevalence (3.6%) suggests possible regional variations in serotype distribution [5].

The incidence observed in this study was lower than rates previously reported in sub-Saharan Africa, such as 210.6 episodes per 1,000 child-years (95% CI: 117.0–355.1) described by Khalil et al. (2018) [5]. This discrepancy is likely attributable to differences in diagnostic methods: while the referenced study used molecular diagnostics, our study relied on stool culture, which is known to be less sensitive. Stool culture’s limitations-including reduced sensitivity due to bacterial viability, sample transport conditions, and selective culture media-likely contributed to an underestimation of the true burden [31,32]. Molecular diagnostics, such as quantitative PCR or RLDT (Rapid diagnostic tests and loop-mediated isothermal amplification method), have demonstrated significantly greater sensitivity, detecting up to twice as many cases as stool culture [33]. Applying a multiplier based on McQuade et al. (2020) [32], suggests the true incidence could be as high as 74.8 cases per 1,000 child-months.

The timing of the study during the peak of the COVID-19 pandemic may have further influenced the findings, as fear of exposure likely reduced health-seeking behavior and contributed to underreporting of diarrheal illnesses. Similar declines in shigellosis incidence were observed in Israel during the pandemic period [34].

Age-specific incidence patterns revealed the highest burden in children aged 12–23 months, with lower incidence during the first year of life-likely due to maternal antibody protection and limited environmental exposure. This age-dependent pattern is consistent with previous studies of *Shigella* infection.

A notable finding is the high incidence of asymptomatic *Shigella* carriage (4.9 per 1,000 child-months; 95% CI: 3.6–6.8). Asymptomatic carriage, often reflecting prolonged post-diarrheal shedding, is a recognized source of ongoing transmission within communities and is associated with adverse outcomes such as stunting, malnutrition, impaired vaccine response, and cognitive deficits [9]. Addressing asymptomatic infections is an urgent need, especially as these carriers may harbor multidrug-resistant strains. Current vaccine development efforts targeting conserved antigens across serotypes could help reduce both symptomatic and asymptomatic infections, improving public health outcomes [15].

*Shigella flexneri* was the most prevalent species, in line with its established role as a major cause of diarrhea in low-income settings with inadequate hygiene and water supply [35]. Preventive measures-such as improved access to safe drinking water and sanitation-remain essential for reducing the burden of shigellosis, as highlighted by studies from Ouagadougou and other regions [16,36].

The strong association between diarrhea and *Shigella* detection (HR=13.5) reinforces *Shigella*’s role as a major diarrheal pathogen. However, the predominance of mucoid (64.8%) over bloody stools (9.1%) challenges traditional dysentery-focused case definitions, suggesting that current protocols may miss many infections. This has important implications for clinical management and surveillance [37].

WASH (Water, Sanitation, and Hygiene) findings from this study were mixed. While public water sources showed borderline protective effects, other WASH indicators were not significantly associated with *Shigella* risk, potentially reflecting high baseline contamination or measurement limitations. The unexpected inverse association between stunting and *Shigella* detection warrants further investigation, possibly related to nutritional impacts on immune response or care-seeking behavior.

Contrary to expectations, regular handwashing among household members was associated with higher odds of *Shigella* detection. This may be due to the use of contaminated water for handwashing, inconsistent soap use, or increased exposure risks in crowded households. These findings highlight the complexity of behavioral and environmental factors influencing *Shigella* transmission. Household crowding, close contact with symptomatic individuals, inadequate sanitation, and poor waste disposal were all correlated with increased incidence. Lower caregiver education and limited hygiene awareness were also associated with higher infection risk.

Several limitations should be considered. First, the focus on a peri-urban area may limit generalizability to rural or urban populations with different socio-environmental conditions [11]. Second, the reliance on stool culture likely underestimated shigellosis incidence compared to more sensitive molecular diagnostics [32,33,38,39]. Third, severe cases requiring hospitalization were not captured, as the study was limited to outpatient settings, potentially missing the full spectrum of disease severity [40]. Fourth, the one-year duration may not capture seasonal or interannual variation in *Shigella* incidence [41]. Additionally, risk factor assessment relied on self-reported data, which is susceptible to recall and social desirability biases, and some variables were only measured at a single time point, possibly missing changes over time.

Despite these limitations, the study has notable strengths. The cohort design enabled collection of both symptomatic and asymptomatic samples, providing a comprehensive estimate of incidence. The combination of active community and passive facility-based surveillance improved case detection, particularly for asymptomatic carriers, and strengthened the reliability of the findings [42]. While less sensitive than molecular methods, stool culture remains valuable for isolating viable pathogens and conducting antimicrobial resistance profiling, which is essential for guiding treatment and informing public health interventions against antimicrobial resistance [43].

In summary, this study highlights the substantial burden of shigellosis among young children in peri-urban Ouagadougou and underscores the need for improved diagnostics, targeted interventions, and continued surveillance to inform effective prevention and control strategies.

**Conclusion**

*Shigella* remains an important cause of moderate to severe diarrhea in children under five in Burkina Faso, confirming its endemicity and public health importance in this setting. Our findings demonstrate an overall incidence rate of 6.8 (95% CI: 5.2, 8.8) per 1000 child-months among this vulnerable population. Among the 56 confirmed incident cases, all four Shigella serogroups were identified, with a clear predominance of *S. flexneri* (58.9%). The remaining circulating serogroups were S. boydii (23.2%), *S. sonnei* (14.3%), and *S. dysenteriae* (3.6%). This serogroup distribution, especially the high prevalence of *S. flexneri*, directly informs vaccine development strategies for Burkina Faso, underscoring the need for a vaccine covering this prevalent serogroup.

These findings underscore the urgent need to strengthen hygiene, sanitation, and safe water storage measures to reduce transmission. Surveillance should be improved, and the adoption of more sensitive diagnostic methods is critical to better assess the true burden of disease, as conventional stool culture likely underestimates incidence due to its limited sensitivity and accessibility in resource-constrained settings. Repeated *Shigella* infections are associated with long-term consequences, including intestinal dysfunction and malnutrition, highlighting the need for accelerated vaccine development and implementation. The high incidence and the predominance of *S. flexneri* strongly suggest that a vaccine targeting this species, particularly a multivalent conjugate vaccine that includes *S. flexneri* serotypes, would be highly relevant and beneficial for reducing the burden of shigellosis in Burkina Faso. Targeted interventions addressing risk factors such as poor hygiene and household overcrowding are essential to reduce *Shigella* transmission. Complete characterization of circulating *Shigella* strains will inform vaccine development strategies and provide valuable data for future vaccine programs. Expanding access to rapid, sensitive diagnostic tools in peripheral health facilities would enable earlier detection and more effective management of shigellosis, ultimately reducing morbidity and mortality among young children in Burkina Faso.

**Acknowledgments**

The authors would like to thank the ShigaPlexIM Consortium members for their contribution to the efficient management of the consortium which is the framework of this study. We also thank the staff of the “Groupe de Recherche Action en Santé (GRAS)”, the community members, and especially the participants in this study.

**Funding**

This research is part of the EDCTP2 programme, supported by the European Union (grant number RIA2018V-2308-ShigaPlexIM). The funders had no role in study design, data collection and analysis, decision to publish, or preparation of the manuscript.

**Competing interests**

The authors have declared that no competing interests exist.

**Author Contributions**

Conceived and designed the experiments: AH, SJ, TBA, AO, SOH, MS. and SBS. Performed the experiments: AH, SJ, SBI, TBA, AO, HD, SOH, MS and SBS. Analyzed the data: OZA, AH and SJ and TBA. Contributed reagents/materials/analysis tools: AH, NI, SJ, SBI, OZA, TBA, MS and SBS. Wrote and review of the paper: AH, SJ, SBI, HD, AD, SSS, OZA, NI, SOH, AO, TBA, MS and SBS.

**References**

1.	Kyu HH, Vongpradith A, Dominguez RMV, Ma J, Albertson SB, Novotney A, et al. Global, regional, and national age-sex-specific burden of diarrhoeal diseases, their risk factors, and aetiologies, 1990–2021, for 204 countries and territories: a systematic analysis for the Global Burden of Disease Study 2021. The Lancet Infectious Diseases. 1 mai 2025;25(5):519‑36.

2.	Anderson M, Sansonetti PJ, Marteyn BS. Shigella Diversity and Changing Landscape: Insights for the Twenty-First Century. Front Cell Infect Microbiol. 2016;6:45.

3.	Nyarkoh R, Odoom A, Donkor ES. Prevalence of Shigella species and antimicrobial resistance patterns in Africa: systematic review and meta-analysis. BMC Infect Dis. 29 oct 2024;24(1):1217.

4.	Liu H, Zhu B, Qiu S, Xia Y, Liang B, Yang C, et al. Dominant serotype distribution and antimicrobial resistance profile of Shigella spp. in Xinjiang, China. PLoS One. 2018;13(4):e0195259.

5.	Khalil IA, Troeger C, Blacker BF, Rao PC, Brown A, Atherly DE, et al. Morbidity and mortality due to shigella and enterotoxigenic Escherichia coli diarrhoea: the Global Burden of Disease Study 1990–2016. The Lancet Infectious Diseases. 1 nov 2018;18(11):1229‑40.

6.	Rogawski McQuade ETR, Scharf RJ, Svensen E, Huggins A, Maphula A, Bayo E, et al. Impact of Shigella infections and inflammation early in life on child growth and school-aged cognitive outcomes: Findings from three birth cohorts over eight years. PLOS Neglected Tropical Diseases. 23 sept 2022;16(9):e0010722.

7.	Berendes D, Capone D, Knee J, Holcomb D, Sultana S, Pickering AJ, et al. Associations between enteric pathogen carriage and height-for-age, weight-for-age and weight-for-height in children under 5 years old in urban Dhaka, Bangladesh. Epidemiol Infect. 27 févr 2020;148:e39.

8.	Luoma J, Adubra L, Ashorn P, Ashorn U, Bendabenda J, Dewey KG, et al. Association between asymptomatic infections and linear growth in 18-24-month-old Malawian children. Matern Child Nutr. janv 2023;19(1):e13417.

9.	Nasrin S, Haque MA, Palit P, Das R, Mahfuz M, Faruque ASG, et al. Incidence of Asymptomatic Shigella Infection and Association with the Composite Index of Anthropometric Failure among Children Aged 1–24 Months in Low-Resource Settings. Life. mai 2022;12(5):607.

10.	Taneja N, Mewara A. Shigellosis: Epidemiology in India. Indian J Med Res. mai 2016;143(5):565‑76.

11.	Badr HS, Colston JM, Nguyen NLH, Chen YT, Burnett E, Ali SA, et al. Spatiotemporal variation in risk of Shigella infection in childhood: a global risk mapping and prediction model using individual participant data. The Lancet Global Health. 1 mars 2023;11(3):e373‑84.

12.	Hausdorff WP, Anderson JD, Bagamian KH, Bourgeois AL, Mills M, Sawe F, et al. Vaccine value profile for *Shigella*. Vaccine. 3 nov 2023;41:S76‑94.

13.	Fleming JA, Gurley N, Knudson S, Kabore L, Bawa JT, Dapaah P, et al. Exploring Shigella vaccine priorities and preferences: Results from a mixed-methods study in low- and middle-income settings. Vaccine X. 9 août 2023;15:100368.

14.	Anderson JD, Bagamian KH, Pecenka CJ, Muhib F, Puett CA, Hausdorff WP, et al. Potential impact and cost-effectiveness of Shigella vaccination in 102 low-income and middle-income countries in children aged 5 years or younger: a modelling study. The Lancet Global Health. 1 juin 2023;11(6):e880‑91.

15.	Lu T, Das S, Howlader DR, Picking WD, Picking WL. Shigella Vaccines: The Continuing Unmet Challenge. International Journal of Molecular Sciences. janv 2024;25(8):4329.

16.	Nitiema LW, Nordgren J, Ouermi D, Dianou D, Traore AS, Svensson L, et al. Burden of rotavirus and other enteropathogens among children with diarrhea in Burkina Faso. Int J Infect Dis. sept 2011;15(9):e646-652.

17.	DuPont HL. Acute Infectious Diarrhea in Immunocompetent Adults. New England Journal of Medicine. 17 avr 2014;370(16):1532‑40.

18.	Rogawski ET, Platts-Mills JA, Seidman JC, John S, Mahfuz M, Ulak M, et al. Use of antibiotics in children younger than two years in eight countries: a prospective cohort study. Bull World Health Organ. 1 janv 2017;95(1):49‑61.

19.	WHO bacterial priority pathogens list, 2024: Bacterial pathogens of public health importance to guide research, development and strategies to prevent and control antimicrobial resistance [Internet]. [cité 17 sept 2025]. Disponible sur: https://www.who.int/publications/i/item/9789240093461

20.	Baker S, Scott TA. Antimicrobial-resistant Shigella: where do we go next? Nature Reviews Microbiology. 15 mai 2023;21(7):409.

21.	Salam MA, Al-Amin MY, Salam MT, Pawar JS, Akhter N, Rabaan AA, et al. Antimicrobial Resistance: A Growing Serious Threat for Global Public Health. Healthcare. janv 2023;11(13):1946.

22.	Ranjbar R, Farahani A. Shigella: Antibiotic-Resistance Mechanisms And New Horizons For Treatment. Infect Drug Resist. 7 oct 2019;12:3137‑67.

23.	Cinquième Recensement Général de la Population et de l’Habitation [Internet]. [cité 22 juill 2022]. Disponible sur: http://www.insd.bf/index.php/publications?id=199

24.	Soura AB, Lankoande B, Millogo R, Bangha M. Comparing causes of death between formal and informal neighborhoods in urban Africa: evidence from Ouagadougou Health and Demographic Surveillance System. Glob Health Action. 2014;7:25523.

25.	Richmond A, Myers I, Namuli H. Urban Informality and Vulnerability: A Case Study in Kampala, Uganda. Urban Science. mars 2018;2(1):22.

26.	Diarrhoeal disease [Internet]. [cité 15 sept 2025]. Disponible sur: https://www.who.int/news-room/fact-sheets/detail/diarrhoeal-disease

27.	Tesfaye TS, Magarsa AU, Zeleke TM. Moderate to Severe Diarrhea and Associated Factors Among Under-Five Children in Wonago District, South Ethiopia: A Cross-Sectional Study. Pediatric Health Med Ther. 19 oct 2020;11:437‑43.

28.	Kotloff KL, Blackwelder WC, Nasrin D, Nataro JP, Farag TH, Eijk A van, et al. The Global Enteric Multicenter Study (GEMS) of Diarrheal Disease in Infants and Young Children in Developing Countries: Epidemiologic and Clinical Methods of the Case/Control Study. Clinical Infectious Diseases: An Official Publication of the Infectious Diseases Society of America. 15 déc 2012;55(Suppl 4):S232.

29.	Jorgensen JH, Carroll KC, Funke G, Pfaller MA, Landry ML, Richter SS, et al., éditeurs. *Manual of* Clinical Microbiology [Internet]. Washington, DC, USA: ASM Press; 2015 [cité 15 sept 2025]. Disponible sur: http://doi.wiley.com/10.1128/9781555817381

30.	WHO child growth standards: length/height-for-age, weight-for-age, weight-for-length, weight-for-height and body mass index-for-age: methods and development [Internet]. [cité 17 févr 2025]. Disponible sur: https://www.who.int/publications/i/item/924154693X

31.	Platts-Mills JA, Liu J, Rogawski ET, Kabir F, Lertsethtakarn P, Siguas M, et al. Use of quantitative molecular diagnostic methods to assess the aetiology, burden, and clinical characteristics of diarrhoea in children in low-resource settings: a reanalysis of the MAL-ED cohort study. Lancet Glob Health. déc 2018;6(12):e1309‑18.

32.	Rogawski McQuade ET, Shaheen F, Kabir F, Rizvi A, Platts-Mills JA, Aziz F, et al. Epidemiology of Shigella infections and diarrhea in the first two years of life using culture-independent diagnostics in 8 low-resource settings. PLoS Negl Trop Dis. août 2020;14(8):e0008536.

33.	Héma A, Sermé SS, Sawadogo J, Diarra A, Barry A, Ouédraogo AZ, et al. Contribution of the Rapid LAMP-Based Diagnostic Test (RLDT) to the Evaluation of Enterotoxigenic Escherichia coli (ETEC) and Shigella in Childhood Diarrhea in the Peri-Urban Area of Ouagadougou, Burkina Faso. Microorganisms. 19 nov 2023;11(11):2809.

34.	Bassal R, Keinan-Boker L, Cohen D. A Significant Decrease in the Incidence of Shigellosis in Israel during COVID-19 Pandemic. Int J Environ Res Public Health. 16 mars 2021;18(6):3070.

35.	Nisa I, Qasim M, Yasin N, Ullah R, Ali A. Shigella flexneri: an emerging pathogen. Folia Microbiol (Praha). avr 2020;65(2):275‑91.

36.	Chiyangi H, Muma JB, Malama S, Manyahi J, Abade A, Kwenda G, et al. Identification and antimicrobial resistance patterns of bacterial enteropathogens from children aged 0–59 months at the University Teaching Hospital, Lusaka, Zambia: a prospective cross sectional study. BMC Infectious Diseases. 2 févr 2017;17(1):117.

37.	Tickell KD, Brander RL, Atlas HE, Pernica JM, Walson JL, Pavlinac PB. Identification and management of Shigella infection in children with diarrhoea: a systematic review and meta-analysis. The Lancet Global Health. 1 déc 2017;5(12):e1235‑48.

38.	Chakraborty S, Connor S, Velagic M. Development of a simple, rapid, and sensitive diagnostic assay for enterotoxigenic E. coli and Shigella spp applicable to endemic countries. PLOS Neglected Tropical Diseases. 28 janv 2022;16(1):e0010180.

39.	Kotloff KL, Nataro JP, Blackwelder WC, Nasrin D, Farag TH, Panchalingam S, et al. Burden and aetiology of diarrhoeal disease in infants and young children in developing countries (the Global Enteric Multicenter Study, GEMS): a prospective, case-control study. The Lancet. 20 juill 2013;382(9888):209‑22.

40.	Acácio S, Mandomando I, Nhampossa T, Quintó L, Vubil D, Sacoor C, et al. Risk factors for death among children 0-59 months of age with moderate-to-severe diarrhea in Manhiça district, southern Mozambique. BMC Infect Dis. 15 avr 2019;19(1):322.

41.	Levine MM, Kotloff KL, Nataro JP, Muhsen K. The Global Enteric Multicenter Study (GEMS): Impetus, Rationale, and Genesis. Clin Infect Dis. 15 déc 2012;55(Suppl 4):S215‑24.

42.	Troeger C, Blacker BF, Khalil IA, Rao PC, Cao S, Zimsen SR, et al. Estimates of the global, regional, and national morbidity, mortality, and aetiologies of diarrhoea in 195 countries: a systematic analysis for the Global Burden of Disease Study 2016. The Lancet Infectious Diseases. 1 nov 2018;18(11):1211‑28.

43.	González-Torralba A, García-Esteban C, Alós JI. Enteropathogens and antibiotics. Enferm Infecc Microbiol Clin (Engl Ed). janv 2018;36(1):47‑54.
