# Supplementary material for: Burden of Shigella infection in children under five years living in a peri-urban area of Ouagadougou, Burkina Faso: A community-based cohort study
Source: PLoS Negl Trop Dis. 2026 Aug 14;20(8):e0014624. doi: 10.1371/journal.pntd.0014624 (PMC13475976; doi:10.1371/journal.pntd.0014624)
Supplement: Supplementary file 3 [file pntd.0014624.s004.docx]

**haveGeneral comments**

**PNTD-D-25-00346_R1**

This manuscript highlighted the burden of *Shigella* infection in peri-urban area Ouagadougou, Burkina Faso among children less than 5 years

The authors have sufficiently addressed and updated the references, recruitment and selection procedure. However, the variables selection process of multivariate analysis and the analysis process are not clear in the analysis section. Furthermore, the results for multivariate analysis have not described in the results and discussion section. The results are not always consistent between univariate and multivariate analysis. So, results and discussion might be changed considering multivariate analysis. These are the major drawback in the resubmitted manuscript. I strongly recommend to address these sections and submit a revised manuscript.

**The following comments are to review and address/update in the manuscript:**

1. Page 4, L-82: Reference number **7-9** did not assess psychomotor development. Please check and update.
2. L111-117: Is any consortium multisite paper published? If yes, give a reference. Would the consortium like to publish site specific manuscript instead of combined paper?
3. Page 9, L 168-169: The line indicated as “trained clinical staff conducted scheduled and unscheduled home visits to collect epidemiological, clinical, and laboratory data throughout” is not very clear. Can you clear about scheduled and unscheduled home visit. Try to make a foot note in Figure 2 to make it clear or in the text
4. P-10. L 179-180: Please give a WHO reference for seven days diarrhoea free for new episode. Give appropriate reference if it is not WHO
5. P-10, L 185-188: The references are not matching with the current case definition. Please check the protocol for case definition of moderate-to-severe diarrhoea or reconfirmed the references for case definition of moderate-to-severe diarrhoea?
6. Page 13, 244-245: Did the investigators analysed only MSD and associated risk factors?
7. Page 13, 249-251: How did the authors assess WAZ, weight for height Z-score for dehydrated children? Is it after correction of dehydration?
8. Page 16, L-291: The range is 2-60 months. However, children less than 5 years were enrolled. The range cannot be upto 60 month.
9. Page 18, Incidence: How many of 236 diarrhoeal episodes had MSD?
10. Page 19, line 320: Indicate table 2 after 1000 child-months
11. Page 20, Line: Shigella incidence is 10.1 in the text and 10.0 in the table among children aged 12-23 months
12. Page 20, L 326-327: describe diarrhea severity classification by Vesicari score in methods section and give baseline data of severity prevalence out of 236 episodes diarrhea cases
13. Page 22, table 2: “Type of toilet into the house” will be likely “Type of improved toilet into the house”. Check/confirm and update
14. Results and discussion: See my earlier comments. The results of age, Socioeconomic status, public water source are different between univariate and multivariate analysis.
15. Page 26, L-380: Distribution of Shigella species with Figure 3 is not matching.
16. Page 26, Figure. Maximum age group will be 59 instead of 60 as per recruitment criteria.
17. P 26, L-386: Heading is “Clinical Characteristics of Shigella diarrhea”. However, contains of the text is not matching with the title.

How shigella diarrhea and shigellosis are defined?

18. Page 27, L 390: P value will be 3 numerical value after decimal point
19. page 27, Table 4. Needs to update. See my comments:

- 230 MSD cases and 230 episode of diarrhea (available data) are confusing and contradictory. Did the protocol only enrol MSD cases? However, manuscript states that 236 diarrheal episodes were recorded
- Table 4 (Title). Similarly, title indicated as MSD cases with Shigella positivity. Furthermore, MSD cases also included Shigella positive cases. The data are overlapping in MSD cases. The denominator should be 212 vs. 18.
- Duplicate presentation of same variables. As for example, Gender (both Male and female, Type of diarrhea (bloody and non-bloody), Clinical features, Nutritional status. Use only ether male or female, bloody diarrhea, fever, stunted, wasted and underweight
- Row 1: delete 7.8%.

20. Discussion:

- The results of multivariate analysis has not been evaluated in discussion section
- L-427- Give reference for the sentence “This age-dependent pattern is consistent with previous studies of *Shigella…..”*
- Line 433: Ref 9 did not assess cognitive deficit
- L 453: I see protective effect in Table 3
- Line 495-501: This is statement from other studies. Better to give references
- The text “Household crowding, close contact with symptomatic individuals, inadequate sanitation, and poor waste disposal were all correlated with increased incidence. Lower caregiver education and limited hygiene awareness were also associated with higher infection risk” does not reflect the study findings. Are this statement from this study or from other studies findings? Give reference if it is from other study finding and discuss with this study findings.
- Current quadrivalent vaccine contains S. Flexner and *S. Sonnei*. Is there any reason *S. Boydii* is high compared to *S. Sonnei* in this setting

21. **Page 33, Conclusion:**

- Update as per earlier comments and try to co-relate with study findings.
- Don’t need to include the results again
