# Supplementary material for: Burden of Shigella infection in children under five years living in a peri-urban area of Ouagadougou, Burkina Faso: A community-based cohort study
Source: PLoS Negl Trop Dis. 2026 Aug 14;20(8):e0014624. doi: 10.1371/journal.pntd.0014624 (PMC13475976; doi:10.1371/journal.pntd.0014624)
Supplement: Supplementary file 4 [file pntd.0014624.s005.docx]

**Response to Editors and Reviewers for Manuscript PNTD-D-25-00346**

Dear Dr. Pascoe, Dr. Wunder, Dr. Kamhawi, Dr. Brindley, and esteemed Reviewers,

We sincerely thank you for the opportunity to revise our manuscript, “Burden of Shigella infection in children under five living in peri-urban area of Ouagadougou, Burkina Faso: A community-based cohort study” (PNTD-D-25-00346). We are grateful for the thorough and constructive feedback provided by the editors and reviewers, which has been invaluable in strengthening our paper. We have carefully considered all comments and have made extensive revisions to the manuscript to address them.

Below, we provide a point-by-point response to the comments. All changes have been incorporated into the revised manuscript, and the specific locations of major revisions are indicated.

**Part A: Response to additional Editor comments**

| **Editor's comment** | **Author's response and action** |
| --- | --- |
| 1. **Objectives & design:** state clear objectives/hypotheses; justify sample size/power; add a map of sampling sites; describe recruitment, inclusion/exclusion, surveillance procedures, and ethical oversight per STROBE. | We have comprehensively addressed these points:   - **Objectives**: the objectives of the study have been more clearly articulated in the final paragraph of the Introduction section. - **Sample Size**: we have added a new subsection, "Sample Size Calculation," to the Methods section to provide a detailed justification for our sample size. - **Map**: a map of the sampling sites has been created and included as Figure 1 in the revised manuscript. - **Recruitment & Procedures**: the "Study design and participants" subsection has been significantly expanded to detail the recruitment process, explicitly list the inclusion and exclusion criteria, and describe surveillance procedures as per STROBE guidelines. |
| 2. **Methods consistency & references:** Reconcile the study period; correct reference mismatches and supply appropriate global-burden and WHO citations; move definitions to the correct sections. | - **Study Period:** we have corrected the inconsistency. The text now clarifies that the study enrolment occurred between December 2020 and March 2021, and each participant was followed for 12 months, with the final follow-up concluding in March 2022 - **References**: we have undertaken a thorough review and correction of all citations. Inappropriate references for the global burden of diarrhoea, WHO guidelines, and others have been replaced with correct and more appropriate sources. - **Definitions:** the "Definitions" subsection has been moved from "Laboratory Methods" to a more appropriate position within the main "Methods" section. |
| 3. **Analysis clarity:** Explain the rationale for using both univariable and multivariable models, variable selection, handling of missing data, and construction of child-months at risk. Ensure tables specify reference categories. | - **The "Statistical analysis**" section now includes a clear rationale for using both univariable and multivariable models. - **Missing data**: we now address the handling of missing data in the "Statistical analysis" section and have noted the reasons for it in the results - **Child-Months Calculation**: we have clarified how the "child-months at risk" denominator was calculated for all participants, including asymptomatic cases and those who did not attend follow-up visits. |
| 4. **Results completeness:** Fill missing table entries, resolve internal inconsistencies (e.g., incidence by severity vs Table 2), correct totals/denominators, clarify Figure 1 legend. | - The missing incidence value for households with "Unimproved sanitation" has been calculated and added to the table. - Incidence inconsistency: The text and Table 2 have been harmonized to ensure the incidence rates by diarrhoea severity are consistent. - Table 1 totals: we have corrected the erroneous counts in Table 1. The total number of participants for each variable now correctly reflects the study population of 750, accounting for missing data. |
| 5. **Public-health relevance:** In Conclusions, explicitly report *Shigella* prevalence and circulating serogroups/serotypes and discuss vaccine implications for Burkina Faso. | The Conclusion has been substantially rewritten. It now explicitly states the key findings on *Shigella* incidence and serogroup distribution (*S. flexneri* being predominant) and directly discusses the implications for vaccine development and public health policy in Burkina Faso. |
| 6. **Data availability:** Deposit de-identified participant-level data, a data dictionary, and analysis code in an open repository and update the Data Availability statement with a working DOI. | Thank you for emphasizing the importance of data transparency and accessibility. We have deposited the fully de-identified participant-level dataset, an accompanying data dictionary, and the complete analysis code into the Open Science Framework (OSF) repository to ensure open access and reproducibility.  The Data Availability statement in the manuscript has been updated to include the permanent, working DOI for this repository: <https://doi.org/10.17605/OSF.IO/EW4UG> |

**Part B: Response to Reviewer #1**

| Reviewer #1's comment | Author's response and action |
| --- | --- |
| 1. **Objectives:** Objectives of the study need to be clearly articulated. | Thank you. As noted in our response to the editor, we have revised the Introduction to more clearly state the study's objectives. |
| 2. **Sample size:** Authors need to figure out how they proceeded to evaluate the size. | We appreciate this point. A new subsection has been added to the Methods explaining the parameters used for the sample size calculation. |
| 3. **Methods:** It's will better to show sampling site on a map in the manuscript. | This is an excellent suggestion. We have created a map illustrating the study area and have included it after the study site section (**Figure 1**). |
| 4. **Methods (*Shigella* identification):** I am really glad to see that strains have been double check in able to confirm that you isolated *Shigella* | Thank you for your positive feedback. Indeed, all *Shigella* isolates were confirmed through double-checking with the BD Phoenix M50 system to ensure accurate species identification and strengthen the validity of our findings. |
| 5. **Analysis:** Why did you perform univariate analysis and multivariate analysis for the risk factors associated with *Shigella* infection in children in the manuscrit? | We appreciate the request for clarification. We have added a sentence in the **"Statistical analysis"** section to explain our rationale: "Bivariate models were first used to screen for potential associations between individual covariates and *Shigella* infection risk. Subsequently, a multivariate model was employed to identify independent risk factors while controlling for the potential confounding effects of other variables." |
| 6. **Results (Table 2):** The incidence of "no" for Type of toilet into the house is absent. | Thank you for catching this omission. We have now calculated this value from our dataset and have added it to **Table 2** (Page 25). |
| 7. **Conclusion:** The conclusion does not highlight the results of the manuscript. There is no mention of the prevalence of *Shigella* and nothing about the different serotypes circulating in Burkina Faso... results of this research should be used to guide public health decisions. | We agree this is an important point. The Conclusion has been extensively revised to lead with our main findings, including the overall incidence (6.8 cases per 1000 child-months) and the predominance of S. flexneri (58.9%), and we now discuss the direct relevance of these findings for future vaccine strategies and public health interventions in the region. |

**Part C: Response to Reviewer #2 (Dr. M. Jahangir Hossain)**

| **Reviewer #2's comment (from attached file)** | Author's response and action |
| --- | --- |
| **Overall: The major drawbacks are mismatched references and unclear recruitment/selection procedures. Recommends to re-write and re-submit.** | We thank the reviewer for this direct and helpful feedback. We have performed a major revision of the manuscript, focusing heavily on correcting all cited references and completely rewriting the Methods section for clarity, as detailed below. |
| 1. **Study Period (Page 2, L 17-18 & Page 7, L 128): The duration of 12 months and the period (Dec 2020-Mar 2022) do not match.** | Thank you for highlighting this inconsistency. The study enrolment period was from December 2020 to March 2021. Each participant was then followed for 12 months, meaning the final follow-up visits concluded in March 2022. We have corrected the text in both the abstract and the main body (Page 8-9, line 145-149) to ensure consistency and clarity. |
| 2. **P-4. L 60: Ref # 1 did not analyze global burden and death. Provide appropriate mortality data and reference.** | We agree and thank the reviewer for this correction. We have replaced the original reference with a more appropriate and authoritative source: The Global Burden of Disease Study 2021 (Kyu et al., Lancet Infect Dis 2025). This reference provides comprehensive global mortality estimates for diarrheal diseases. The text and citation have been updated accordingly. |
| 3. **P-4, line 63: The reference # 2 is not appropriate. This paper (ref#) represented data from sub-Saharan Africa.** | We thank the reviewer for this observation. The cited reference (Demissie et al.) was indeed specific to sub-Saharan Africa. We have replaced it with the Global Burden of Disease Study 2021 (Kyu et al., Lancet Infect Dis 2025), which provides the global context for the statement that South Asia and sub-Saharan Africa account for the majority of diarrheal deaths in children. |
| 4. **P-4, Line 69-70: Give a reference** | Thank you. We have added supporting references for the geographical distribution of Shigella serogroups. We now cite Anderson et al. (2016) for the classical stratification and a recent meta-analysis by Nyarkoh et al. (2024) confirming the continued predominance of S. flexneri in Africa. |
| 5. **P-4, Line 72: Ref # 4 does not reflect global data** | We agree. The original reference (Liu et al., 2018) was a local study from China. We have replaced it with the recent meta-analysis by Nyarkoh et al. (2024), which provides a broader, more representative analysis of *Shigella* prevalence and serotype distribution in Africa, making it a more suitable global reference for this context. |
| 6. **P-4, Line 73: Cite appropriate reference and the text** | we have updated the sentence and its citation. The statement “Shigellosis is one of the most prevalent diarrheal diseases in developing countries” is now supported by the comprehensive African meta-analysis by Nyarkoh et al. (2024) (<https://pmc.ncbi.nlm.nih.gov/articles/PMC11520789/>). |
| 7. **Line 76-77 (Ref 6, 3, 7): provide appropriate citations.** | We have updated these citations. The impact of shigellosis on psychomotor development is now supported by Rogawski McQuade et al. (2022), and the role of asymptomatic carriage is supported by Luoma et al. (2023) and Nasrin et al. (2022). |
| 8. **Page 5, L 99: Find appropriate reference.** | We have updated the reference to the most recent and authoritative source: the WHO Bacterial Priority Pathogens List (2024) (https://www.who.int/publications/i/item/9789240093461), which explicitly classifies Shigella as a priority pathogen due to antimicrobial resistance, underscoring the need for new interventions. We also cite a recent comprehensive review on antimicrobial-resistant Shigella detailing resistance patterns and challenges: https://pmc.ncbi.nlm.nih.gov/articles/PMC10184058/. |
| 9. **Page 7, L 128: Check the time period with abstract and make consistent** | We thank the reviewer for raising this important point concerning the consistency of the study period. To address this, we have thoroughly reviewed and revised the manuscript to ensure alignment between the abstract and main text. The revised text now clearly states:  "Between December 2020 and March 2021, participants under five years of age were recruited from a peri-urban community in Ouagadougou. Follow-up of the last enrolled participant concluded in March 2022." |
| 10. **Page 7 (Study design and participants): The enrolment procedures of study participants are not clear. What were the criteria to be part of the study and to be excluded from the study? What was the population census/demographic characteristics in the study area? Where was the child enrolled? I see screening (page 9, line 158) was done at the health centre.** | We thank the reviewer for this critical feedback. We have completely rewritten this section to provide a detailed, step-by-step description of:   - Inclusion/exclusion criteria: clearly defined (e.g., children <5 yrs, permanent residents, informed consent; exclusion for immunodeficiencies, etc.). - Recruitment process: detailed the community mobilization, household census by community health workers, creation of volunteer lists, and the quota-based random selection process from these lists. - Study area demographics: added a brief description of the population characteristics based on local census data. - Enrollment location: specified that enrollment and baseline (M0) visits occurred at the two designated health centres (Polesgo CSPS and Kossodo CMA) |
| 11. **Page 8, Line: Explain more details about parent education** | We have clarified that parent education was achieved through community sensitization campaigns. These activities informed parents and guardians about diarrhoea symptoms and stressed the importance of seeking care promptly at the designated sentinel health centres. |
| 12. **Page 8, line-145-158: Definitions are not the part of laboratory method** | We agree. We have moved the "Definitions" subsection to appear immediately after "Study Design and Participants" and before the "Laboratory Methods" section, ensuring a more logical flow. |
| 13. **Page 8, line 149-150: How was “after a period” without diarrhea or dysentery defined?** | Thank you for requesting this clarification. The phrase “after a period” has been explicitly defined as “a minimum of 7 consecutive days without diarrhea or dysentery,” which is consistent with WHO guidelines and standard epidemiological practice for defining new episodes. |
| 14. **Page 8, line 148: The ref # 25 is not WHO guidelines** | we apologize for this error. We have replaced reference #25 with the correct citation: the WHO diarrhoeal disease fact sheet (<https://www.who.int/news-room/fact-sheets/detail/diarrhoeal-disease>), which provides the standard definition of diarrhoea. |
| 15. **Page 8, line 156: Is it standard reference for moderate-to-severe diarrhea?** | We have updated the reference to better support the definition. The criteria for Moderate-to-Severe Diarrhea (MSD) align with those used in the Global Enteric Multicenter Study (GEMS). We now cite Tesfaye et al. (2020), who applied this specific GEMS/WHD-defined case definition in a study among under-five children. |
| 16. **Page 9, Stool Samples collection: How stool samples were collected in health facility from asymptomatic cases? There are also concern to collect stool samples from diarrhea cases** | We have added detail to clarify the process:   - Asymptomatic children: parents were provided with sterile containers and instructions to collect a sample at home before scheduled visits or at the health facility upon arrival. - Diarrheal cases: fresh stool samples were collected by trained health personnel at the health centre at the time of presentation, prior to any treatment, to ensure sample quality. |
| 17. **Page 169, ref 27: Is the ref # 27 appropriate?** | We have updated this reference (now #29) to an authoritative source on clinical microbiology procedures: The Manual of Clinical Microbiology, 11th Edition (Jorgensen et al., 2015), which is a standard reference in the field. |
| 18. **Page 10-11, L 198-201: Give reference** | thank you. We have added the correct citation for the WHO Child Growth Standards (2006) to the paragraph describing the calculation of Z-scores and the definition of undernutrition (available at: <https://www.who.int/publications/i/item/924154693X>) |
| 19. **Page 11: How total child-month at risk was calculated for asymptomatic cases or who did not attend in sentinel health centres after an episode of diarrhea?** | we have clarified in the Methods section that child-months at risk were calculated for all children from enrolment until their first Shigella-positive sample or until censoring (due to loss to follow-up or study end). This includes time when children were asymptomatic and periods between health centre visits. Person-time was aggregated across the entire cohort to calculate incidence rates. |
| 20. **Page 11, L-219: ref # 28 is not WHO growth standards.** | We apologize for this error. We have replaced reference #28 with the correct citation: WHO Child Growth Standards (2006). Available at: <https://www.who.int/publications/i/item/924154693X> |
| 21. **Page 13, Table 1: Make number of numerical value consistent after decimal point.** | We have revised Table 1 to ensure all numerical values are consistent to one decimal place. |
| 22. **Page 13, Table 1: A trained research team collected data. However, missing values for mother’s age, education, occupation, Marital status, Mother’s number of children, socioeconomic status, main source of drinking water, handwashing habit and type of toilet were high.**   **Total enrolled: 750 children. However, total number for mother age: 214+536+71=821** | We thank the reviewer for their meticulous check. The total of 821 was an error in manuscript preparation; the value "71" was incorrectly entered. Mother's age data is complete for all 750 participants with no missing values. We have corrected Table 1. For other variables with legitimate missing data due to non-response, this is now clearly indicated in the table footnote. We apologize for the confusion. |
| 23. **Page 16, Figure 1. Elaborate for “M”. How will the investigators explain for 750 children present in “M0” visit? How were they invited to participate in the study?** | We have revised the figure (now it is figure 2) legend and the Methods text to clarify:  This flowchart illustrates participant enrollment and stool sample collection during a twelve-month cohort study in peri-urban Ouagadougou, Burkina Faso. A total of 750 children were enrolled and provided non-diarrheal stool samples at baseline (Month 0), Month 6, and Month 12. Passive case detection identified 236 diarrheal episodes from 181 children during follow-up. In total, 2,401 stool samples (236 diarrheal and 2,165 non-diarrheal) were collected and tested for Shigella using culture methods. |
| 24. **Page 16, L 256-257: Please explain clearly incidence by diarrhea severity. The incidence are not matching with table 2 for moderate and severe cases by Vesikari score** | Thank you for catching this discrepancy. The values in the text were incorrect. We have corrected the text to report the accurate incidence rates shown in Table 2: 28.7 per 1000 child-months for moderate cases and 47.2 per 1000 child-months for severe cases. |
| 25. **Page 19 & 21, line 271, table 4: What were the reference value for toilet type** | The reference category for "Toilet type" is "Improved sanitation." We have made this explicit in the table footnote for clarity. The hazard ratios shown are for the comparison between unimproved and improved sanitation. |
| 26. **Page 24, line 323-324: How the percentage of vomiting (3.4%) and percentage of mucoid stool (8.1%) were calculated?** | We apologize; this sentence was incorrect and poorly phrased. We have rewritten it for clarity, directly referencing the correct calculations from Table 3: *"*Shigella* positivity was not significantly associated with vomiting (6.9% vs. 8.5% in those without, p=0.661) or the presence of mucoid stools (9.4% vs. 8.1% in those without, p=0.120)."* |
| 27. **Page 26, L 343: Ref # 29 is not related with culture, molecular diagnostics and sensitivity** | We agree and thank the reviewer. We have removed the incorrect WHO growth standards reference and replaced it with Rogawski McQuade et al. (2020), a key study that directly compares and demonstrates the superior sensitivity of molecular diagnostics (qPCR) over traditional culture for detecting *Shigella*. |
| 28. **Page 26-27: check and update appropriate ref.** | We have verified and confirm that the citation to Bassal et al. (2021) is appropriate, as it documents the significant decline in shigellosis incidence in Israel during the COVID-19 pandemic, supporting our discussion point. |
