# Supplementary material for: Burden of Shigella infection in children under five years living in a peri-urban area of Ouagadougou, Burkina Faso: A community-based cohort study
Source: PLoS Negl Trop Dis. 2026 Aug 14;20(8):e0014624. doi: 10.1371/journal.pntd.0014624 (PMC13475976; doi:10.1371/journal.pntd.0014624)
Supplement: Supplementary file 5 [file pntd.0014624.s006.docx]

**Rebuttal letter**

**Manuscript Number**: PNTD-D-25-00346

**Title**: Burden of *Shigella* infection in children under five living in peri-urban area of Ouagadougou, Burkina Faso: A community-based cohort study

**Journal**: PLOS Neglected Tropical Diseases

**Dear Dr. Pascoe and the Editorial Board,**

We sincerely thank you for the opportunity to revise our manuscript. We are grateful for the thorough and constructive feedback provided by the reviewers, which has been invaluable in strengthening our paper. We have carefully considered all comments and have made extensive revisions to the manuscript to address them, particularly regarding the alignment of our results and discussion with the multivariate analysis.

Below is a point-by-point response to the reviewer’s comments.

**Response to Reviewer #1**

**General Comments**: *The authors have sufficiently addressed and updated the references. However, the variables selection process of multivariate analysis and the analysis process are not clear... Furthermore, the results for multivariate analysis have not been described in the results and discussion section... I strongly recommend addressing these sections.*

Response: we sincerely thank the reviewer for identifying this critical gap. We agree that the multivariate analysis represents our most robust findings and should be the focal point of the manuscript. Action: We have extensively rewritten the "Risk Factors" subsection of the Results and the corresponding paragraphs in the Discussion. The text now clearly highlights the significant multivariate findings (specifically regarding age, socioeconomic status, and public water sources). We have also clarified the variable selection process in the Methods section.

1. **Page 4, L-82: Reference number 7-9 did not assess psychomotor development. Please check and update.**

Response: You are correct. References 7–9 focus on growth and anthropometric failure, while Reference 6 discusses cognitive outcomes. Action: We have revised the sentence: "In these children, shigellosis can impair psychomotor development [6] and contribute to linear growth faltering [7–9], even in asymptomatic carriers."

2. **L111-117: Is any consortium multisite paper published?**

Response: a combined multisite paper is not currently planned. Due to COVID-19 logistical challenges and technical reagent limitations at the Burkina Faso site, the consortium decided to publish site-specific manuscripts. Action: We have added a reference to the related molecular analysis (RLDT) performed on a subset of these samples (Héma A, et al. Microorganisms 2023, 11, 2809).

3. **Page 9, L 168-169: Clarify scheduled and unscheduled home visits.**

Response: We apologize for the confusion. All visits were conducted at health facilities; no home visits were performed. Action: We corrected the text and added a footnote to Figure 2 defining "Scheduled visits" (planned monthly surveillance) and "Unscheduled visits" (passive surveillance for diarrheal symptoms).

4. **P-10. L 179-180: Give a reference for the 7-day diarrhea-free interval.**

Response: we followed the GEMS methodology to ensure the independence of episodes. Action: We replaced the generic citation with the GEMS methodological reference: Kotloff KL, et al. Clin Infect Dis. 2012;55(Suppl 4):S232–S245.

5. **P-10, L 185-188: Case definition of Moderate-to-Severe Diarrhea (MSD).**

Response: Our MSD definition (dehydration, dysentery, or hospitalization) is adapted from GEMS. Action: The text was revised to explicitly list these criteria, supported by the Kotloff et al. (2012) reference.

6. **Page 13, 244-245: Did the investigators analyze only MSD and associated risk factors?**

Response: Incidence and risk factor analysis included all confirmed Shigella events (symptomatic and asymptomatic). Only the clinical characteristics table focused on the diarrheal subset. Action: Clarified in Methods and Results.

7. **Page 13, 249-251: Anthropometric assessment and dehydration.**

Response: Baseline Z-scores (WAZ, HAZ, WHZ) were calculated using measurements from the enrollment visit, where all children were healthy and free of diarrhea. Action: Added a clarifying statement to the Methods: "Nutritional status was assessed at enrollment while children were asymptomatic; Z-score calculations were not impacted by acute dehydration."

8. **Page 16, L-291: Correction of age range.**

Action: Corrected to "0–59 months" throughout the text and figures.

9. **Page 18, Incidence: How many of 236 diarrheal episodes had MSD?**

Response: 225 out of 236 episodes were classified as moderate or severe. Action: Results updated: "Based on the Vesikari scoring system, 225 (95.3%) of these episodes were classified as Moderate-to-Severe Diarrhea (MSD)..."

10. **Page 19, line 320: Indicate table 2 after 1000 child-months**

Action: Inserted "(Table 2)"

11. **Page 19-20: Table 2 references and rounding error.**

Corrected the incidence rate for 12-23-month-olds to 10.0 to match the table.

12. **Page 20, L 326-327: describe diarrhea severity classification by Vesicari score in methods section and give baseline data of severity prevalence out of 236 episodes diarrhea cases**

**Action: we have added a description of the Vesikari Clinical Severity Score to the Methods (evaluating duration, intensity, dehydration). We have also included the breakdown of the 236 episodes by severity in the Results text.**

Action: we have added a description of the Vesikari Clinical Severity Score to the Methods (evaluating duration, intensity, dehydration). We have also included the breakdown of the 236 episodes by severity in the Results text.

13. **Page 22, table 2: “Type of toilet into the house” will be likely “Type of improved toilet into the house”. Check/confirm and update**

Renamed the sanitation variable to "Improved Sanitation" in Table 2.

14. **Results and Discussion: Multivariate vs. Univariate findings.**

Response: we agree that the multivariate results are the most important. Action: We revised the Results and Discussion to emphasize that access to public water sources was significantly protective (HR: 0.5, p=0.031), while age 12–23 months was the highest risk group (HR: 2.7).

15. **Page 26, L-380: Distribution of Shigella species with Figure 3 is not matching.**

Action: Re-verified species counts (S. flexneri 58.9%, S. boydii 23.2%, etc.) and updated the figure bars/labels to match.

16. **Figure 3 (now Figure 4) and Age labels.**

Corrected age x-axis to "48–59 months."

17. **P 26, L-386: Heading is “Clinical Characteristics of Shigella diarrhea”. However, contains of the text is not matching with the title.**

**How shigella diarrhea and shigellosis are defined?**

Action: renamed heading to "Clinical Characteristics of Diarrheal Episodes." Standardized all p-values to 3 decimal places.

18. **Page 27, L 390: P value will be 3 numerical values after decimal point**

Response: we have completely redesigned Table 4 to remove overlapping data. Action: The denominator is now 212 (Shigella-) vs. 18 (Shigella+). Duplicate rows (e.g., Male/Female) were removed to show only one category for clarity. Deleted the "7.8%" from the header row.

19. **Page 27, Table 4 Reconstruction.**

Response: we have completely redesigned Table 4 to remove overlapping data. Action: The denominator is now 212 (Shigella-) vs. 18 (Shigella+). Duplicate rows (e.g., Male/Female) were removed to show only one category for clarity. Deleted the "7.8%" from the header row.

20. **Discussion: Evaluation of Multivariate Results. Action:**

- Discussed the protective effect of public water (aHR 0.5).
- Added GEMS citation for age-dependent patterns.
- Removed unsupported statements regarding crowding/education that were not significant in our model.
- Added a discussion on the high prevalence of S. boydii and its implications for quadrivalent vaccine coverage.

21. **Conclusion Update.**

Action: Rewritten to specifically highlight the findings regarding public water sources and age-specific risks (12–23 months) as primary targets for intervention.

*We believe these revisions have significantly improved the manuscript and address all concerns raised by the reviewers. We look forward to your decision.*

*Sincerely,*

Alimatou Héma On behalf of the authors
