# Supplementary material for: Burden of Shigella infection in children under five years living in a peri-urban area of Ouagadougou, Burkina Faso: A community-based cohort study
Source: PLoS Negl Trop Dis. 2026 Aug 14;20(8):e0014624. doi: 10.1371/journal.pntd.0014624 (PMC13475976; doi:10.1371/journal.pntd.0014624)
Supplement: Peer review history

# Peer review: Decision Letter 0

**DOI:** 10.1371/journal.pntd.0014624.r001

**Authors:** Ben Pascoe, Elsio Wunder

11 Aug 2025

Burden of Shigella infection in children under five living in peri-urban area of Ouagadougou, Burkina Faso: A community-based cohort study.

PLOS Neglected Tropical Diseases

Dear Dr. Héma,

Thank you for submitting your manuscript to PLOS Neglected Tropical Diseases. After careful consideration, we feel that it has merit but does not fully meet PLOS Neglected Tropical Diseases's publication criteria as it currently stands. Therefore, we invite you to submit a revised version of the manuscript that addresses the points raised during the review process.

Please submit your revised manuscript within 60 days Oct 10 2025 11:59PM. If you will need more time than this to complete your revisions, please reply to this message or contact the journal office at plosntds@plos.org. When you're ready to submit your revision, log on to https://www.editorialmanager.com/pntd/ and select the 'Submissions Needing Revision' folder to locate your manuscript file.

Please include the following items when submitting your revised manuscript:

* A rebuttal letter that responds to each point raised by the editor and reviewer(s). You should upload this letter as a separate file labeled 'Response to Reviewers'. This file does not need to include responses to any formatting updates and technical items listed in the 'Journal Requirements' section below.

* A marked-up copy of your manuscript that highlights changes made to the original version. You should upload this as a separate file labeled 'Revised Manuscript with Track Changes'.

* An unmarked version of your revised paper without tracked changes. You should upload this as a separate file labeled 'Manuscript'.

If you would like to make changes to your financial disclosure, competing interests statement, or data availability statement, please make these updates within the submission form at the time of resubmission. Guidelines for resubmitting your figure files are available below the reviewer comments at the end of this letter.

We look forward to receiving your revised manuscript.

Kind regards,

Ben Pascoe

Academic Editor

PLOS Neglected Tropical Diseases

Elsio Wunder Jr

Section Editor

PLOS Neglected Tropical Diseases

Shaden Kamhawi

co-Editor-in-Chief

PLOS Neglected Tropical Diseases

orcid.org/0000-0003-4304-636XX

Paul Brindley

co-Editor-in-Chief

PLOS Neglected Tropical Diseases

orcid.org/0000-0003-1765-0002

**Additional Editor Comments (if provided):**

Please address the following essential items:

- Objectives & design: State clear objectives/hypotheses; justify sample size/power; add a map of sampling sites; describe recruitment, inclusion/exclusion, surveillance procedures, and ethical oversight per STROBE.
- Methods consistency & references: Reconcile the study period (e.g., “12 months” vs Dec 2020–Mar 2022) across Abstract/Methods; correct reference mismatches and supply appropriate global-burden and WHO citations; move definitions to the correct sections.
- Analysis clarity: Explain the rationale for using both univariable and multivariable models, variable selection, handling of missing data, and construction of child-months at risk (including asymptomatic and non-attenders). Ensure tables specify reference categories (e.g., toilet type).
- Results completeness: Fill missing table entries (e.g., “No” for toilet type), resolve internal inconsistencies (e.g., incidence by severity vs Table 2), and correct totals/denominators (e.g., Table 1 counts exceeding N). Clarify Figure 1 legend (e.g., “M0”) and all abbreviations.
- Public-health relevance: In Conclusions, explicitly report Shigella prevalence and circulating serogroups/serotypes and discuss vaccine implications for Burkina Faso.
- Data availability: Deposit de-identified participant-level data, a data dictionary, and analysis code in an open repository and update the Data Availability statement with a working DOI. (Reviewer 2 marked data availability as not met.)

**Journal Requirements:**

If the reviewer comments include a recommendation to cite specific previously published works, please review and evaluate these publications to determine whether they are relevant and should be cited. There is no requirement to cite these works unless the editor has indicated otherwise.

[Note: HTML markup is below. Please do not edit.]

**Reviewers' Comments:**

Reviewer's Responses to Questions

**Key Review Criteria Required for Acceptance?**

As you describe the new analyses required for acceptance, please consider the following:

**Methods**

-Are the objectives of the study clearly articulated with a clear testable hypothesis stated?

-Is the study design appropriate to address the stated objectives?

-Is the population clearly described and appropriate for the hypothesis being tested?

-Is the sample size sufficient to ensure adequate power to address the hypothesis being tested?

-Were correct statistical analysis used to support conclusions?

-Are there concerns about ethical or regulatory requirements being met?

Reviewer #1: I congratulate authors on initiating this important study dealing with the burden of Shigella infection in children under five living in peri-urban area of Ouagadougou, Burkina Faso, but I think that objectives of the study need to be clearly articulated.

- Sample size is enough but authors need to figure out how they procceded to evaluated the size

- It's will better to show sampling site on a mapp in the manuscrit.

- I am realy glade to see that strains have been double check in able to confirme that you isolated Shigella

- Why did you performed univariate analysis and multivariate analysis for the risk factors associated with Shigella infection in children in the manuscrit?

Reviewer #2: Objectives of study: OK

Study design and methods: Not mentioned clearly in study design and methods section

Ethical consideration: OK

**********

**Results**

-Does the analysis presented match the analysis plan?

-Are the results clearly and completely presented?

-Are the figures (Tables, Images) of sufficient quality for clarity?

Reviewer #1: Table 2, the incidence of "no" for Type of toilet into the house is absent

Reviewer #2: Results: Presented well. However, there are some gaps. Please see in comments document

**********

**Conclusions**

-Are the conclusions supported by the data presented?

-Are the limitations of analysis clearly described?

-Do the authors discuss how these data can be helpful to advance our understanding of the topic under study?

-Is public health relevance addressed?

Reviewer #1: The conclusion does not highlight the results of the manuscript. There is no mention of the prevalence of Shigella and nothing about the different serotypes circulating in Burkina Faso. I feel that this is important, especially in a context where we are witnessing the development of a vaccine against shigella, and the results of this research should be used to guide public health decisions in this context.

Reviewer #2: Conclusion: OK. However, methods section is not clear

**********

**Editorial and Data Presentation Modifications?**

Use this section for editorial suggestions as well as relatively minor modifications of existing data that would enhance clarity. If the only modifications needed are minor and/or editorial, you may wish to recommend “Minor Revision” or “Accept”.

Reviewer #1: Authors must clearly articulated objectives of the stuy, they must also selected the importants results (serotypes, the important risk factors associated to the burden of Shigella) to prensent and disccussed them in the manuscrit.

Reviewer #2: Please see in my comments. The manuscript could be re-write and re-submit

**********

**Summary and General Comments**

Use this section to provide overall comments, discuss strengths/weaknesses of the study, novelty, significance, general execution and scholarship. You may also include additional comments for the author, including concerns about dual publication, research ethics, or publication ethics. If requesting major revision, please articulate the new experiments that are needed.

Reviewer #1: (No Response)

Reviewer #2: This manuscript highlighted the burden of Shigella infection in peri-urban area Ouagadougou, Burkina Faso among children less than 5 years

The paper has described the result section well. However, there is limitation in background and methods section. The major drawback of this paper are that 1) the references and text are not matching or the references have not been cited properly, and 2) the recruitment and selection procedures of study are not clear or not described in methods section.

Overall recommendation: Re-write and re-submit the manuscript

**********

PLOS authors have the option to publish the peer review history of their article (what does this mean?). If published, this will include your full peer review and any attached files.

If you choose “no”, your identity will remain anonymous but your review may still be made public.

**Do you want your identity to be public for this peer review?**  For information about this choice, including consent withdrawal, please see our Privacy Policy.

Reviewer #1: No

Reviewer #2: **Yes:** M. Jahangir Hossain

[NOTE: If reviewer comments were submitted as an attachment file, they will be attached to this email and accessible via the submission site. Please log into your account, locate the manuscript record, and check for the action link "View Attachments". If this link does not appear, there are no attachment files.]

**Figure resubmission:**

While revising your submission, please upload your figure files to the Preflight Analysis and Conversion Engine (PACE) digital diagnostic tool, https://pacev2.apexcovantage.com/. PACE helps ensure that figures meet PLOS requirements. To use PACE, you must first register as a user. Registration is free. Then, login and navigate to the UPLOAD tab, where you will find detailed instructions on how to use the tool. If you encounter any issues or have any questions when using PACE, please email PLOS at figures@plos.org. Please note that Supporting Information files do not need this step. If there are other versions of figure files still present in your submission file inventory at resubmission, please replace them with the PACE-processed versions.

**Reproducibility:**

Attachment Submitted filename: PNTD-D-25-00346_Reviewers comments_JH.docx

# Author comment: Author response to Decision Letter 1

**DOI:** 10.1371/journal.pntd.0014624.r002

9 Oct 2025

Attachment Submitted filename: Rebuttal_Letter_Response_to_Reviewers_07OCT2025.docx

# Peer review: Decision Letter 1

**DOI:** 10.1371/journal.pntd.0014624.r003

**Authors:** Ben Pascoe, Elsio Wunder

26 Jan 2026

Response to Reviewers Revised Manuscript with Track Changes Manuscript

Shaden Kamhawi

co-Editor-in-Chief

PLOS Neglected Tropical Diseases

orcid.org/0000-0003-4304-636XX

Paul Brindley

co-Editor-in-Chief

PLOS Neglected Tropical Diseases

orcid.org/0000-0003-1765-0002

**Additional Editor Comments (if provided):**

**Journal Requirements:**

**Reviewers' comments:**

**Key Review Criteria Required for Acceptance?**

As you describe the new analyses required for acceptance, please consider the following:

**Methods**

-Are the objectives of the study clearly articulated with a clear testable hypothesis stated?

-Is the study design appropriate to address the stated objectives?

-Is the population clearly described and appropriate for the hypothesis being tested?

-Is the sample size sufficient to ensure adequate power to address the hypothesis being tested?

-Were correct statistical analysis used to support conclusions?

-Are there concerns about ethical or regulatory requirements being met?

Reviewer #1: (No Response)

Reviewer #2: Methods sections have been sufficiently address. However, the variables selection process of multivariate analysis and the analysis process are not clear in the analysis section.

**********

**Results**

-Does the analysis presented match the analysis plan?

-Are the results clearly and completely presented?

-Are the figures (Tables, Images) of sufficient quality for clarity?

Reviewer #1: (No Response)

Reviewer #2: The results has been presented well. Please see my comments. Furthermore, the results for multivariate analysis have not described in the results and discussion section. The results are not always consistent between univariate and multivariate analysis. So, results and discussion might be changed considering multivariate analysis. These are the major drawback in the resubmitted manuscript.

**********

**Conclusions**

-Are the conclusions supported by the data presented?

-Are the limitations of analysis clearly described?

-Do the authors discuss how these data can be helpful to advance our understanding of the topic under study?

-Is public health relevance addressed?

Reviewer #1: (No Response)

Reviewer #2: This is not typically co-related with study findings. The conclusion section needs to revise and update

**********

**Editorial and Data Presentation Modifications?**

Use this section for editorial suggestions as well as relatively minor modifications of existing data that would enhance clarity. If the only modifications needed are minor and/or editorial, you may wish to recommend “Minor Revision” or “Accept”.

Reviewer #1: Manuscript need to be thoroughly proofread and aligned with standards for writing bacterial name and presenting certain statistical results. I made some comments on the Review file attached

Reviewer #2: The manuscript needs revision and to submit a revised manuscript

**********

**Summary and General Comments**

Use this section to provide overall comments, discuss strengths/weaknesses of the study, novelty, significance, general execution and scholarship. You may also include additional comments for the author, including concerns about dual publication, research ethics, or publication ethics. If requesting major revision, please articulate the new experiments that are needed.

Reviewer #1: (No Response)

Reviewer #2: The authors have sufficiently addressed and updated the references, recruitment and selection procedure. However, the variables selection process of multivariate analysis and the analysis process are not clear in the analysis section. Furthermore, the results for multivariate analysis have not described in the results and discussion section. The results are not always consistent between univariate and multivariate analysis. So, results and discussion might be changed considering multivariate analysis. These are the major drawback in the resubmitted manuscript. I strongly recommend to address these sections and submit a revised manuscript.

**********

PLOS authors have the option to publish the peer review history of their article (what does this mean?). If published, this will include your full peer review and any attached files.

If you choose “no”, your identity will remain anonymous but your review may still be made public.

**Do you want your identity to be public for this peer review?**  For information about this choice, including consent withdrawal, please see our Privacy Policy.

Reviewer #1: No

Reviewer #2: **Yes:** M. Jahangir Hossain

**Figure resubmission:**

**Reproducibility:**

To enhance the reproducibility of your results, we recommend that authors of applicable studies deposit laboratory protocols in protocols.io, where a protocol can be assigned its own identifier (DOI) such that it can be cited independently in the future. Additionally, PLOS ONE offers an option to publish peer-reviewed clinical study protocols. Read more information on sharing protocols at https://plos.org/protocols?utm_medium=editorial-email&utm_source=authorletters&utm_campaign=protocols

Attachment Submitted filename: Revise_Manuscript_with_tracking_changes_Comment Reviewer 1.docx

Attachment Submitted filename: PNTD-D-25-00346_R1_Reviewers comments.docx

# Author comment: Author response to Decision Letter 2

**DOI:** 10.1371/journal.pntd.0014624.r004

19 Jun 2026

Attachment Submitted filename: Rebuttal_Letter_Response_to_Reviewers_16FEB2026.docx

# Editor report: Decision Letter 2

**DOI:** 10.1371/journal.pntd.0014624.r005

**Authors:** Franklin Toapanta, Elsio Wunder

29 Jul 2026

Dear Dr Héma,

We are pleased to inform you that your manuscript 'Burden of Shigella infection in children under five years living in a peri-urban area of Ouagadougou, Burkina Faso: A community-based cohort study.' has been provisionally accepted for publication in PLOS Neglected Tropical Diseases.

Before your manuscript can be formally accepted you will need to complete some formatting changes, which you will receive in a follow up email. A member of our team will be in touch with a set of requests.

Please note that your manuscript will not be scheduled for publication until you have made the required changes, so a swift response is appreciated.

IMPORTANT: The editorial review process is now complete. PLOS will only permit corrections to spelling, formatting or significant scientific errors from this point onwards. Requests for major changes, or any which affect the scientific understanding of your work, will cause delays to the publication date of your manuscript.

Should you, your institution's press office or the journal office choose to press release your paper, you will automatically be opted out of early publication. We ask that you notify us now if you or your institution is planning to press release the article. All press must be co-ordinated with PLOS.

Thank you again for supporting Open Access publishing; we are looking forward to publishing your work in PLOS Neglected Tropical Diseases.

Best regards,

Franklin R. Toapanta, M.D. Ph.D.

Academic Editor

PLOS Neglected Tropical Diseases

Elsio Wunder Jr

Section Editor

PLOS Neglected Tropical Diseases

Shaden Kamhawi

co-Editor-in-Chief

PLOS Neglected Tropical Diseases

orcid.org/0000-0003-4304-636XX

Paul Brindley

co-Editor-in-Chief

PLOS Neglected Tropical Diseases

orcid.org/0000-0003-1765-0002

***********************************************************

The authors have addressed the minor issues requested; hence, I am pleased to the authors that the manuscript can be accepted for publication.

p.p1 {margin: 0.0px 0.0px 0.0px 0.0px; line-height: 16.0px; font: 14.0px Arial; color: #323333; -webkit-text-stroke: #323333}span.s1 {font-kerning: none

# Editor report: Acceptance letter

**DOI:** 10.1371/journal.pntd.0014624.r006

**Authors:** Franklin Toapanta, Elsio Wunder

Dear Dr Héma,

We are delighted to inform you that your manuscript, "Burden of Shigella infection in children under five years living in a peri-urban area of Ouagadougou, Burkina Faso: A community-based cohort study.," has been formally accepted for publication in PLOS Neglected Tropical Diseases.

We have now passed your article onto the PLOS Production Department who will complete the rest of the publication process. All authors will receive a confirmation email upon publication.

The corresponding author will soon be receiving a typeset proof for review, to ensure errors have not been introduced during production. Please review the PDF proof of your manuscript carefully, as this is the last chance to correct any scientific or type-setting errors. Please note that major changes, or those which affect the scientific understanding of the work, will likely cause delays to the publication date of your manuscript. Note: Proofs for Front Matter articles (Editorial, Viewpoint, Symposium, Review, etc...) are generated on a different schedule and may not be made available as quickly.

Soon after your final files are uploaded, the early version of your manuscript will be published online unless you opted out of this process. The date of the early version will be your article's publication date. The final article will be published to the same URL, and all versions of the paper will be accessible to readers.

For Research Articles, you will receive an invoice from PLOS for your publication fee after your manuscript has reached the completed accept phase. If you receive an email requesting payment before acceptance or for any other service, this may be a phishing scheme. Learn how to identify phishing emails and protect your accounts at https://explore.plos.org/phishing.

Thank you again for supporting open-access publishing; we are looking forward to publishing your work in PLOS Neglected Tropical Diseases.

Best regards,

Shaden Kamhawi

co-Editor-in-Chief

PLOS Neglected Tropical Diseases

Paul Brindley

co-Editor-in-Chief

PLOS Neglected Tropical Diseases
